# Synthesis and evaluation of L-arabinose-based cationic glycolipids as effective vectors for pDNA and siRNA *in vitro*

**DOI:** 10.1371/journal.pone.0180276

**Published:** 2017-07-03

**Authors:** Bo Li, Wanrong Guo, Fan Zhang, Meiyan Liu, Shang Wang, Zhonghua Liu, Shuanglin Xiang, Youlin Zeng

**Affiliations:** 1 National & Local Joint Engineering Laboratory for New Petro-chemical Materials and Fine Utilization of Resources, College of Chemistry and Chemical Engineering, Hunan Normal University, Changsha, Hunan, P. R. China; 2 Key Laboratory of Protein Chemistry and Developmental Biology of State Education Ministry of China, College of Life Sciences, Hunan Normal University, Changsha, Hunan, P. R. China; 3 The National &Local Joint Engineering Laboratory of Animal Peptide Drug Development, College of Life Sciences, Hunan Normal University, Changsha, Hunan, P.R. China; Universidad de Castilla-La Mancha, SPAIN

## Abstract

Glycolipids might become a new type of promising non-viral gene delivery systems because of their low cytotoxicity, structural diversity, controllable aqua- and lipo-solubility, appropriate density and distribution of positive charges, high transfer efficiency and potential targeting function. In this study, four kinds of L-arabinose-based cationic glycolipids (Ara-DiC12MA, Ara-DiC14MA, Ara-DiC16MA and Ara-DiC18MA) containing quaternary ammonium as hydrophilic headgroup and two alkane chains as hydrophobic domain were synthesized and characterized. They were observed to have strong affinities for plasmid DNA (pDNA) and siRNA, the pDNA can be completely condensed at N/P ratio less than 2, and the siRNA can be completely retarded at N/P ratio less than 3. The dynamic light scattering (DLS) experiment and atomic force microscopy (AFM) experiment demonstrated that cationic lipids and their lipoplexes possessed suitable particle sizes with near-spherical shape and proper ζ-potentials for cell transfection. The Ara-DiC16MA liposome was found to have good transfection efficacy in HEK293, PC-3 and Mat cells compared with other three kinds of liposomes, and also maintain low cytotoxicity and better uptake capability *in vitro*. Furthermore, the gene silencing assay showed that Ara-DiC14MA and Ara-DiC16MA liposomes have demonstrated effective delivery and higher gene knockdown activity (>80%) in the above mentioned cells than Lipofectamine 2000. These results indicated Ara-DiC16MA can be developed for efficient and low toxic gene delivery.

## Introduction

The gene therapy, using various foreign nucleic acids, containing plasmid DNA (pDNA), small interfering RNAs (siRNAs) and oligonucleotides[1–5] to down or unregulated the expression of defective genes, is a promising strategy for treatment of various inherited or acquired diseases, such as AIDS[6, 7], immunodeficiency[8, 9], degenerative disorders[10] and cancer[11, 12]. Gene therapy has been attracted great attention in the field of biomedical sciences and benefited to develop the human health level over the past few decades. A success of gene therapy is required to design and develop a high efficient gene delivery systems to overcome the obstacles in different stages [13, 14]. In the past decades, a lot of viral and non-viral vector systems were designed and developed to delivery foreign nucleic acids [15–17]. Viral vectors including retrovirus vectors, lentivirus vectors, adenovirus vectors and adeno-associated virus vectors [18], are superior in efficient delivery of genetic material into cells *in vivo*, and were widely employed in early clinical trials [19, 20]. However, some side effects including host immune and inflammatory reactions, oncogenic transformation by unpredicted, potential to form replication-competent virions and pathogenic risks of the viral vectors severely limited their applications [21–26]. Recently, non-viral delivery systems were keenly explored to avoid the potential problems meet in viral vectors [27]. However, several hurdles and major trafficking barriers, such as effective cellular uptake capability [28], DNA protection and escaped from acidic vesicles (for example, endosomes and lysosomes) [29, 30], nuclear transport targetability to specific cell types and infection of non-dividing cells, need to be solved during constructing an efficient non-viral delivery systems [31, 32].

In general, non-viral vectors can be divided into three main categories: cationic liposomes, polymers and peptides [33]. Among the non-viral vectors, cationic liposome has many advantages to overcome above mentioned problems, which has excellent potential as gene delivery agents [34, 35]. Since the first cationic liposome, *N*-[1-(2,3-dioleyloxy)propyl]-*N*,*N*,*N*-trimethylammonium chloride (DOTMA), was discovery by Felgner et al.[36], a number of similar systems have been developed subsequently [37, 38]. Chemical components of cationic liposome involve backbone, cationic (hydrophilic) headgroup, hydrophobic tail and linker between above parts, which played vital roles in transfection efficiency. The relationship between the function and structure of liposomes were well investigated and reviewed [39, 40]. Guided by the structure-function relationship, lots of biodegradable and commercial available chemicals such as amino acids [41], oligopeptide [42], cholesterol [43, 44], cyclen [45] and carbohydrates [46, 47] were commonly selected as the backbone of cationic lipids to improve the transfection efficiency and targeting delivery. In recently, more and more carbohydrates-based cationic lipids [48], were designed and synthesized for the reason that carbohydrates are involved in many important physiological processes, including inflammatory and immunological responses, tumor metastasis, cell-cell signaling, apoptosis, cell adhesion, bacterial and viral recognition, and anticoagulation [49]. In addition, carbohydrates are specific ligands to targeting cell surface receptors, which receive significant attention in recent decades. Receptor-mediated endocytosis is promoted to cellular uptake capacity in gene delivery [50]. The ligands corresponding to cell surface receptors including glucose [48, 51], galactose [50, 52], mannose[53], hyaluronic acid (HA) [54] and so on, which have been conjugated in gene delivery vectors.

**Fig 1:**
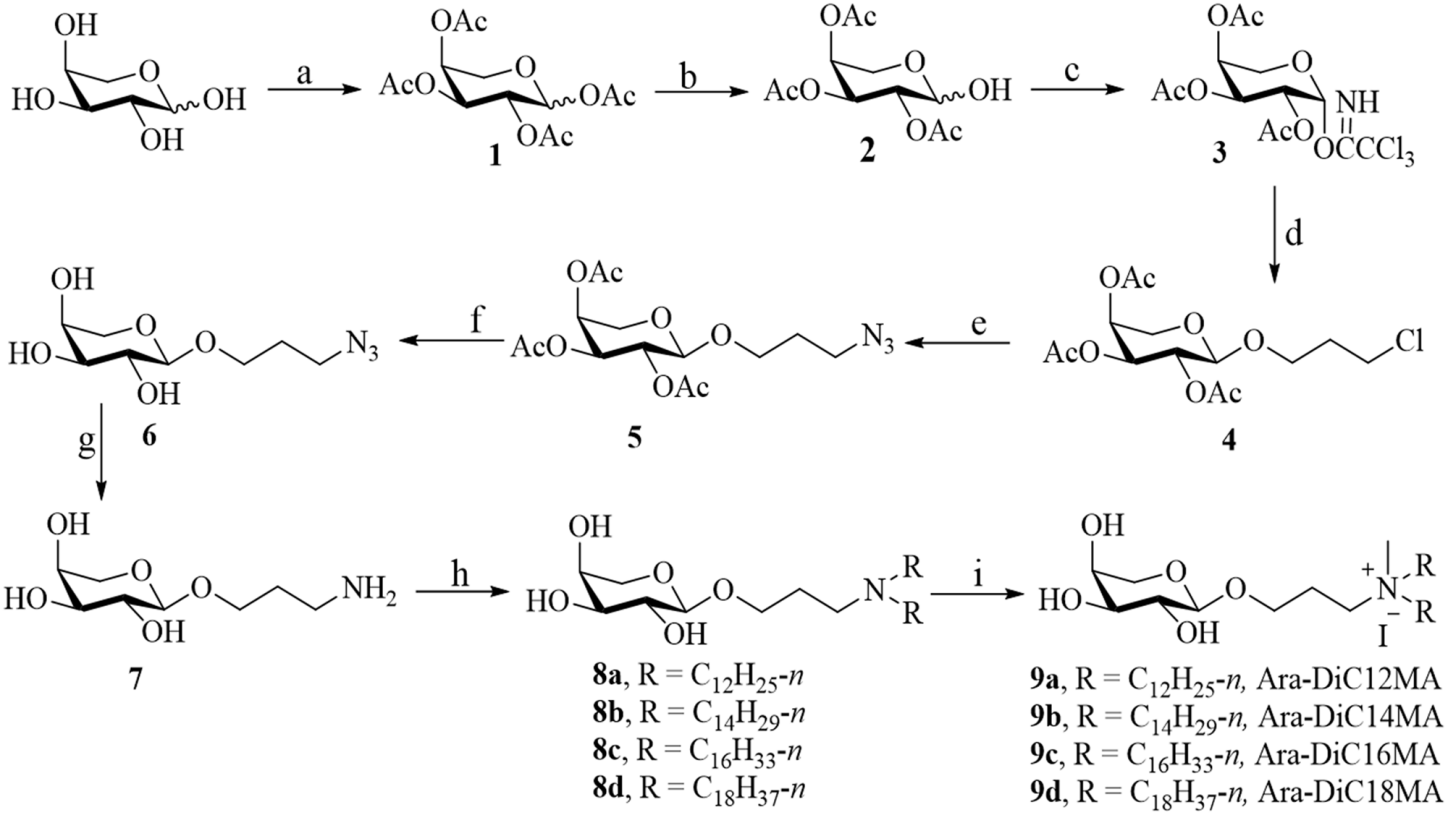
Synthesis of derivatives of arabinose-based cationic liposomes. Reagents and conditions: (a) Ac_2_O, HClO_4_; (b) piperazine, THF; (c) NCCCl_3_, K_2_CO_3_, CH_2_Cl_2_; (d) 3-Chloro-1-propanol, TMSOTf, CH_2_Cl_2_, -20°C to rt; (e) NaN_3_, DMF, 80°C; (f) NH_3_ / CH_3_OH; (g) Ph_3_P, THF, 75°C, reflux; (h) K_2_CO_3_, Alkyl Bromide, C_2_H_5_OH, CH_3_OH, 70°C; (i) CH_3_I, THF.

Along this direction, we devote L-arabinose-based glycolipids to delivery for pDNA and siRNA *in vitro*. Four cationic lipids (Fig 1, Ara-DiC12MA, Ara-DiC14MA, Ara-DiC16MA and Ara-DiC18MA) with different hydrophobic chain lengths were constructed by using L-arabinose as starting material, via peracetylation, selective1-*O*-deacetylation, trichloroacetimidation, glycosylation, azidation, deacetylation, Staudinger reaction, tertiary amination and quaternization. The structures of these synthesized lipids and some intermediates were characterized by ^1^H NMR, ^13^C NMR, ^1^H-^1^H COSY, ^1^H-^13^C HMQC and ESI-MS. Then the four cationic lipids were fabricated to form corresponding liposomes by ultrasonic method. The size and zeta of liposomes, lipid/pDNA complexs, and lipid/siRNA complexs were evaluated by dynamic light scattering (DLS). The morphology of liposomes and lipoplexes were characterized by atomic force microscopy (AFM). The affinity with pDNA and siRNA were measured by gel electrophoresis assay. The transfection efficiency of these liposomes was evaluated by observing enhanced green fluorescent protein (EGFP) expression from pDNA in HEK293, Mat, PC-3, HepG2, MCF-7 and HeLa cells. Furthermore, gene silencing efficiency and cellular uptake capability were assessed in HEK293, Mat and PC-3 cells. Meanwhile, the cytotoxicity of cationic lipids/pDNA complexes were established on all transfected cell lines using MTT assay.

## Experimental section

### Materials

L-arabinose, alkyl bromide, 3-chloro-1-propanol, Triphenylphosphine, iodomethane were purchased from Sinopharm Chemical Reagent Co.,Ltd and Shanghai Bangcheng Chemical Co. Ltd. Dimethyl sulfoxide (DMSO) and methyl thiazol tetrazolium (MTT) were purchased from Sigma-Aldrich (St. Louis, MO). Ethidium bromide, Agarose and Water-DEPC treate (Molecular Biology Grade) were ordered from Sangon Biotech (Shanghai, China). RMPI 1640 medium, F12K, Dulbecco's modified Eagle's medium (DMEM) and Fetal bovine serum (FBS) were purchased from GIBCO (Gaithersburg, MD). Label IT^®^ Tracker^™^ intracellular nucleic acid localization kit including TransIT^®^-LT1 transfection reagent was purchased from Mirus Bio LLC, USA. Hochest 33342 and Dio were purchased from the Beyotime Institute of Biotechnology (Haimen, China). Lipofectamine2000 was obtained from Invitrogen (Carlsbad, USA). Dual-Luciferase Reporter Assay System kit and Dual-Luciferase Report Gene (pMIR-glo) were purchased from Promega Corp. siRNA (antisense: 5’-GUCCAUCAAUAUCAGCUACUU-3’, sense: 5’-GUAGCUGAUAUUGAUGGAC-3’) was purchased from TAKARA Corp. Hek293(Human embryonic kidney cell line), PC-3(human prostate cancer cell line), Mat(Mouse prostate cancer cell line, Mat-Ly-Lu-B-2), HepG2(Human hepatocellular liver carcinoma cell line), MCF-7(human breast adenocarcinoma cell line), HeLa(Human cervical carcinoma cell line) cells and EGFP-C1 Plasmid DNA(pDNA) were donated from School of Life Sciences, Hunan Normal University (Hunan, China). All other chemicals were of analytical grade and were used without further purification. The structures of these synthesized lipids and some intermediates were characterized by ^1^H NMR, ^1^H-^1^H COSY, ^1^H-^13^C HMQC (Bruker 500MHz), ^13^C NMR (Bruker 125MHz) and ESI-MS.

### Cell culture

Hek293, Mat, HepG2 and HeLa cell were maintained in DMEM medium containing 10% (v:v) FBS, 100 units/mL penicillin and 100 mg/mL streptomycin. PC-3 cell was cultured in F12K medium supplemented with 10% FBS. MCF-7 cell was grown in RMPI 1640 medium with 10% FBS and 1% Penicillin-Streptomycin Solution (100×). All the cell lines were cultured in a 95% humidified atmosphere containing 5% CO_2_ at 37°C.

### Liposome and lipoplexes preparation

The cationic lipids were dissolved in sterile double distilled water and then sonicated to clarity at 37°C for 30 min in a closed vial, and filtered with syringe filter (0.45 μm) subsequently to give the solution of cationic liposomes. The concentration of liposome was 0.25 mmol L^-1^ for all lipids (**9a**-**9d**). Liposome/pDNA complexes were formulated by adding 50 μL of Opti-MEM with 0.8 μg of pDNA to give pDNA aliquots. Liposomes were diluted in Opti-MEM^®^ to get lipids aliquots at different N/P ratios (2:1, 4:1, 6:1, 8:1, 10:1). pDNA aliquot was added to the microcentrifuge tubes containing the diluted liposomes, and mixtures were pipetted thoroughly. The lipoplexes were incubated for 30 min at room temperature. Simultaneously, the complexes of liposome/siRNA and Lipofectamine2000/pMIR-glo were prepared similar to Liposome/pDNA lipoplexes as described above.

### Agarose gel electrophoresis experiments

A gel retardation assay was performed to examine the condensation ability of liposomes to pDNA and siRNA by electrophoresis. Liposome/pDNA and liposome/siRNA complexes were prepared at different N/P ratios ranging from 0.3 to 3 with 1.2 μg pDNA and 0.4 μg siRNA (30 pmol), respectively. Naked pDNA and siRNA were used as a control. The Liposome/pDNA complexes were electrophoresed (DYY-6C, Beijing Liuyi Biotechnology Co. Ltd) on 1% agarose gel in tris-borate-EDTA buffer at 100 mA for 20 min, and the Liposome/siRNA was electrophoresed on 4% agarose gel at 40 mA for 15 min. pDNA and siRNA retardation were visualized by staining with ethidium bromide (EB) under a UV illuminator (Tanon 2500, Shanghai Tianneng science and Technology Co. Ltd).

### Morphology study by atomic force microscopy (AFM)

The morphology of cationic lipids and corresponding pDNA or siRNA complexes were executed by atom force microscopy. The samples were prepared by dropping cationic lipids or complexes solutions (prepared as described above) onto fresh mica, and air-dried at room temperature prior to AFM measurements. Then, image was performed on Nanoscope IV atomic force microscope (Veeco Instruments) at room temperature.

### Particle size and zeta potential measurements

Liposome/pDNA or siRNA complexes with N/P ratios of at 2:1, 4:1, 6:1, 8:1 and 10:1 were prepared in double distilled water as subscripted in section of Liposome and lipoplexes preparation. The size and zeta potential of liposome, and liposome/pDNA or siRNA complexes were measured by dynamic light scattering system (Zetasizer Nano ZS90, Malvern Instruments led.,UK) at 25°C.

### Cellular uptake of liposome/pDNA complexes

In order to evaluate the cellular uptake capacity of the different liposome/pDNA complexes, Hek293, Mat, PC-3 and HeLa cells were seeded with the proper density of cells in laser confocal culture dish and cultivated at 37°C with 5% CO_2_ overnight. The liposomes/Cy3-labeled pDNA complexes were prepared by mixing liposomes and Cy3-labeled EGFP pDNA at different N/P charge ratios followed by incubating at room temperature for 30 min. Before transfection, medium was exchanged with fresh basic medium. Then the complexes solutions were added to the culture dish and transfected for 4 h. After that the cells were washed with phosphate buffered saline (PBS, 0.5×) for three times, Dio (excitation at 484 nm and emission at 501 nm) was used to label the cytomembrane (green) for 20 min, and the Hochest 33342 (excitation at 346 nm and emission at 460 nm) was used to stain the nuclei (blue) for 15 min. Prior to fluorescence microscopic imaging, cells were washed three times with PBS buffer to eliminate the background signals. Afterward, images were observed and recorded by Nikon Ti-U invert fluorescence microscope.

### Expression of the enhanced green fluorescent protein (EGFP) gene

Hek293, HeLa, Mat, PC-3, MCF-7 and HepG-2 cells were seeded in 24-well plates and cultured overnight to reach an appropriate cell density. Before gene transfection, the plate was washed with PBS and the medium was exchanged with serum-free medium. Afterwards, the liposome/pDNA complexes containing 0.8 μg plasmid DNA (prepared as described in section of Liposome and lipoplexes preparation) was added to each well. After 4 h of incubation, the medium was changed to 10% FBS fresh complete growth medium. The green fluorescent protein was detected under an inverted fluorescence microscope (Olympus LX71) after 24 h incubation. For the quantitative assay, the cells were measured with Cellometer K2 imager cytometer (Nexcelom Bioscience LLC, USA). The transfection efficiency was defined as the percentage of GFP positive cells per total tested cells, and the positive control treated with Lipofectamine2000/pDNA was converted to 100%.

### *In vitro* gene silencing

Hek293, Mat and PC-3 cells were seeded with proper density in 48-well plates and incubated for 24 h. Before transfection, medium was exchanged with 250 μL fresh basic medium when cells reached a 50–60% density. Then liposome/siRNA and Lipofectamine2000/pMIR-glo complexes (noteworthy, pMIR-glo can expressing firefly luciferase and renal luciferase) solutions were added to the plate successively and transfected for 4 h. Meanwhile, the Lipofectamine2000/siRNA was set as positive control. Thereafter, the culture medium was again replaced with fresh complete growth medium and further incubated for another 24 h. Then, the total protein of cells was then collected was analyzed according to the protocol of Dual-Luciferase Reporter Assay System kit. And relative luciferase activity was measured by Luminometer (TD-20/20, Turner Designs).

### *In vitro* cytotoxicity assay

Cell viability was analyzed by MTT (3-(4,5-dimethyl-2-thiazolyl)-2,5-diphenyl-2-H-tetrazoli -um bromide) assay as previously described. Hek293, HeLa, Mat, PC-3, MCF-7 and HepG-2 cells were incubated in 96 well plates with 10% FBS fresh complete growth medium for 24 h at 37°C under 5% CO_2_. The cells were then treated with lipoplexes (prepared as described section in Liposome and lipoplexes preparation). After incubation for 4 h, 20μL of MTT (5 mg/mL) was added to each well and the cells was incubated further for 4 h. The medium was then removed, and 150 μL of DMSO was added to each well for dissolution of the formed purple formazan crystals. After shaking for 20 min, absorbance values were measured at 490 nm in a microplate reader (SpectraMax i3x, USA), and normalized to that obtained by untreated control cells. The % viability was then calculated as [{A490(treated cells) − background]/[A490(untreated cells) − background}] × 100.

### Statistical analysis

All results are reported as mean ± standard deviation (SD). Statistical variation *p* values between group means were calculated by Student’s *t*-test. Date with *p* values of less than 0.05 was considered statistically significant.

## Results and discussion

### Synthesis and characterization of L-arabinose-based cationic glycolipids

As shown in Fig 1, four arabinose-based cationic lipids **9a-9d** (labeled as Ara-DiC12MA, Ara-DiC14MA, Ara-DiC16MA Ara-DiC18MA respectively) were synthesized. Briefly, under the catalysis of perchloric acid, all the hydroxyls of L-arabinose were masked with acetyl to give compound **1**, which was transferred into glycosylation donor **3** by selective 1-O-deacetylation with piperazine in tetrahydrofuran, followed by trichloroacetimidation with trichloroacetonitrile in the presence of potassium carbonate in anhydrous dichloromethane. Light yellow syrup **4** was accomplished by glycosylation of donor **3** with 3-chloro-1-propanol in the presence of TMSOTf. Then, the significant intermediate **6** was furnished from compound **4** by azidation with sodium azide, and subsequently deacetylation with saturated ammonia in methonal-THF. Then the amine **7** was constructed from compound **6** by the Staudinger reaction. The dialkylmethylamine **8** was prepared through coupling between compound **7** and alkyl bromine in alcohol and potassium carbonate. The target compounds **9a-9d** could be obtained by simple quaternization in tetrahydrofuran with iodomethane. The structures of target compounds and some synthetic intermediates were characterized by ^1^H NMR, ^13^C NMR, ^1^H-^1^H COSY, ^1^H-^13^C HMQC and ESI-MS. The detail procedures for synthesis lipids **9a-9d** and NMR spectrums were included in the Supporting Information.

### Determination of pDNA or siRNA affinity by agarose gel electrophoresis

**Fig 2:**
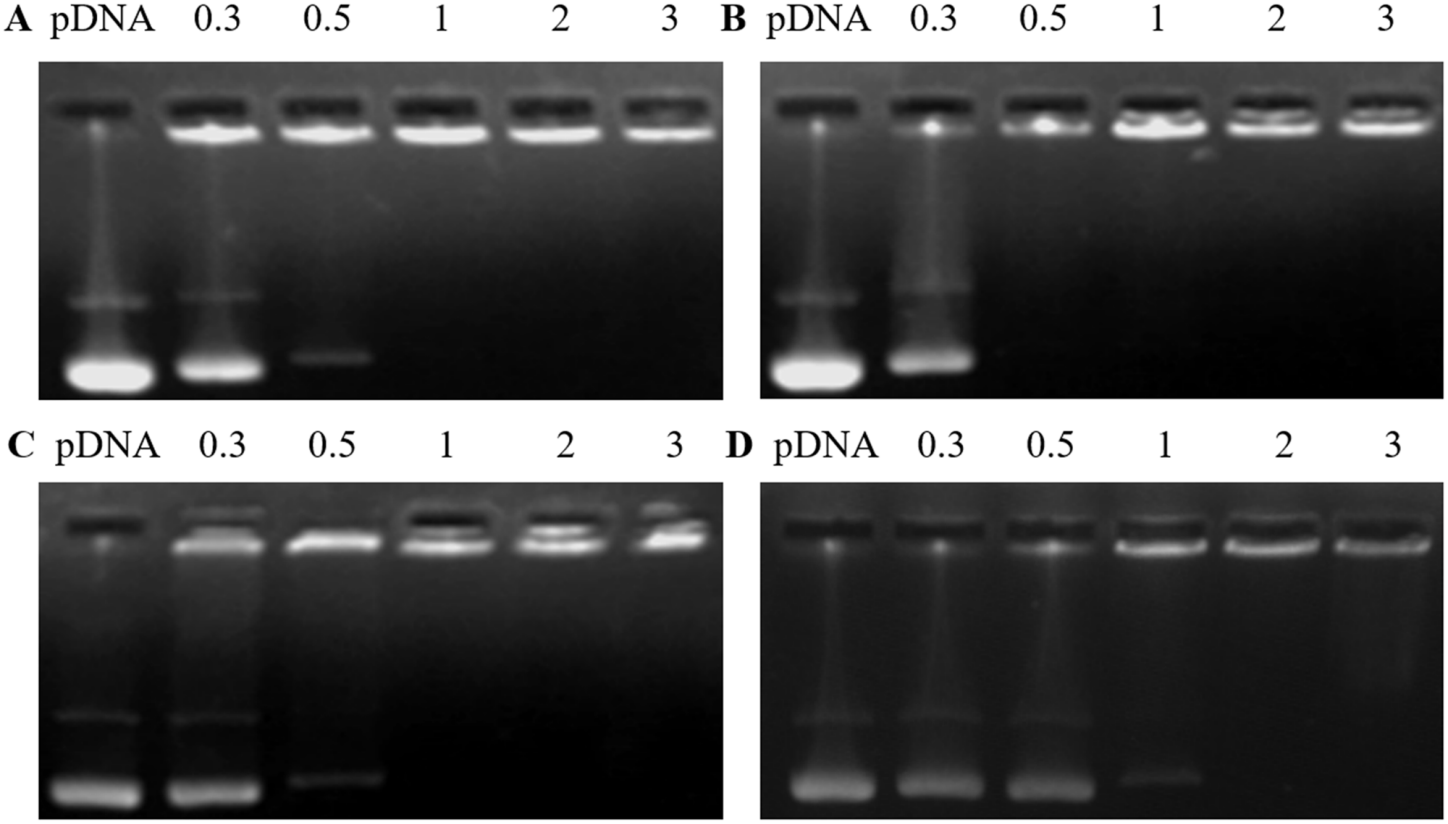
Determination of pDNA affinity by agarose gel electrophoresis. Gel electrophoresis assay of cationic liposome Ara-DiC12MA/pDNA (A, **9a**), Ara-DiC14MA/pDNA (B, **9b**), Ara-DiC16MA/pDNA (C, **9c**), Ara-DiC18MA/pDNA (D, **9d**) complexes with pDNA at N/P ratios of 0.3:1, 05:1, 1:1, 2:1 and 3:1, respectively.

Agarose gel electrophoresis experiments were used to estimate the pDNA or siRNA binding ability of the cationic liposomes. pDNA and siRNA were combined with cationic liposomes of Ara-DiC12MA, Ara-DiC14MA, Ara-DiC16MA, Ara-DiC18MA liposomes at the N/P ratios of 0.3:1, 0.5:1, 1:1, 2:1, 3:1. As positive control, naked pDNA was also applied for the agarose gel electrophoresis experiments. As shown in Fig 2, the pDNA was successfully retarded by Ara-DiC14MA and Ara-DiC18MA lipoplexes at the N/P ratios of 0.5:1 (Fig 2B) and 2:1 (Fig 2D), respectively. The mobility of pDNA with cationic liposomes Ara-DiC12MA and Ara-DiC16MA were prohibited at the N/P ratios of 1:1(Fig 2A and 2C). The result indicated that the four liposomes have strong pDNA-binding ability and can completely condensed pDNA at an N/P ratio less than 2. Meanwhile, the electrophoresis experiments also showed that the movement of siRNA (S2 Fig) at N/P ratio of 0.5:1 was entirely retarded except Ara-DiC18MA (retarded at the N/P ratios of 3:1).

### Particle size, zeta potential and morphology measurements

**Fig 3:**
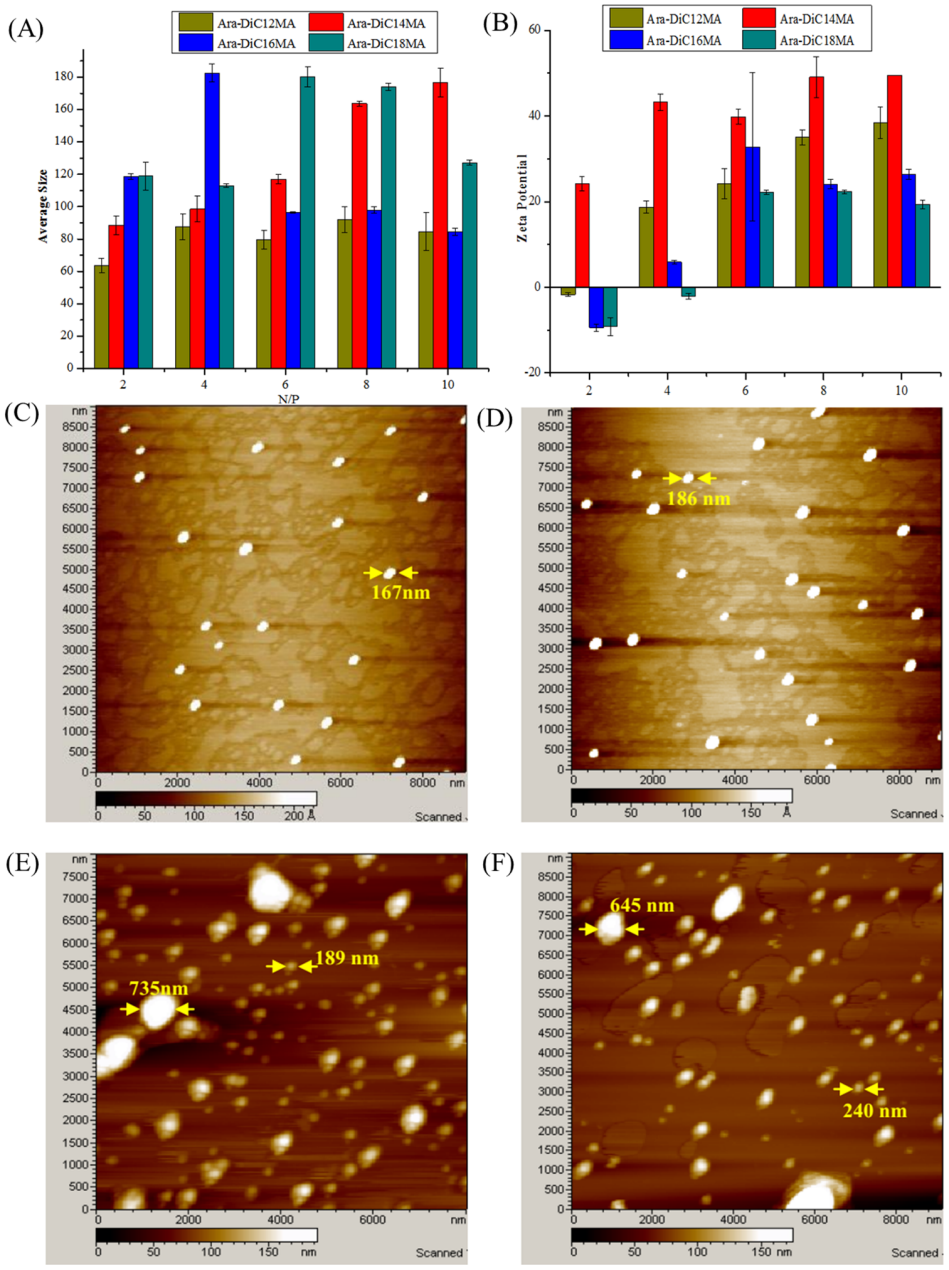
Particle size, zeta potential and morphology measurements. (A) Mean particle size and (B) zeta potential of the lipoplexes formed from each lipid at various liposomes/pDNA ratios. AFM morphologies images of the naked lipids Ara-DiC14MA lipid (C), Ara-DiC16MA lipid (D) and the lipoplexes of Ara-DiC14MA/pDNA (E), Ara-DiC16MA/pDNA (F) at N/P ratio of 6. The scale bar is Z Axis Color Scale. Each value represents the mean ± standard deviation of three measurements.

It is well known that the apparent cell toxicity, the cellular uptake and release of non-viral vectors strongly depend on physicochemical properties of lipsome/lipoplexes including particle size, zeta potential and morphology [55, 56]. The dynamic light scattering (Table 1 and Fig 3A) showed that the average particle sizes of Ara-DiC12MA, Ara-DiC14MA, Ara-DiC16MA and Ara-DiC18MA liposomes are 135 nm, 136 nm, 148 nm and 111.8 nm respectively, and the average sizes of pDNA-complexes vary from 63 nm to 180 nm. It was observed that the size of naked liposomes increased with the increase in the length of saturated alkyl chain except Ara-DiC18MA. However, no noticeable trend was observed after liposome/pDNA complexes were formed at different N/P ratios. The low polydispersity index (PDI), as a measure of homogeneity, revealed the uniform size distribution in naked liposomes and lipoplexes (Table 1 and S1 Table). According to the conclusion from literature [57], the average particle sizes of these liposomes and lipoplexes were suitable for gene delivery. In addition, the positive surface potentials of the prepared lipoplexes are essential for intracellular uptake [58]. As shown in Table 1, the zeta potential of four naked liposomes are between 50 and 70 mV, which ensured condensation of negatively charged pDNA via electrostatic interaction. According to the Fig 3B, the Ara-DiC18MA lipoplexes exhibited a positively charged surface at the N/P ratios of 6:1. The Ara-DiC12MA and Ara-DiC16MA lipoplexes became positively charged at N/P ratios higher than 2:1. Nevertheless, Ara-DiC14MA always maintain positively charged under various lipoplexes N/P ratios (2:1–10:1). The physicochemical data mentioned above also suggested that the Ara-DiC12MA, Ara-DiC14MA and Ara-DiC16MA liposomes form stable lipoplexes more effectively than Ara-DiC18MA.

**Table 1:** The average size and zeta potential of cationic liposomes 9a-9d.

Cationic liposomes	Average size (nm)	PDI	zeta potential(mV)
**9a**, Ara-DiC12MA	135.0±3.5	0.234±0.027	+65.4±0.1
**9b**, Ara-DiC14MA	136.0±0.7	0.212±0.006	+70.3±0.7
**9c**, Ara-DiC16MA	148.0±2.7	0.150±0.010	+53.2±0.8
**9d**, Ara-DiC18MA	111.8±1.2	0.175±0.018	+50.4±0.6

The average size of siRNA-liposomes complexes varies from 187 nm to 475 nm, which is slightly larger than naked liposome and liposomes/pDNA complexes (S2 Table and S1 Fig). It may due to the less change of siRNA (-3.1 mV) than pDNA (-11.9 mV), which lead to different micelles aggregation. Notably, the Ara-DiC16MA/siRNA complex process similar sizes around 190 nm and is slightly smaller than other lipoplexes. Furthermore, all complexes always maintain positively charged at various N/P ratios (2:1–10:1) (S1 Fig).

The morphology of the liposomes, liposome/pDNA and liposome/siRNA complexes formed from Ara-DiC14MA, Ara-DiC16MA were examined by atom force microscopy (AFM). As shown in Fig 3, the AFM images of naked liposomes formed from Ara-DiC14MA lipid (Fig 3C) and Ara-DiC16MA lipid (Fig 3D) appeared to be near-spherical shape with average size around 200 nm. When pDNA-complexes and siRNA-complexes were formed, the morphology of complexes was still near-spherical shape, however, the average size enlarged variously (Figs 3E, 3F, S1E and S1F) and the homogeneity got worse.

As a whole, the chain length of the hydrophobic domain determines the fluidity of the bilayer and influences the stability of liposomes [59]. When the liposomes have assembled into lipoplexes with pDNA or siRNA by electrostatic interaction, the characterization (such as particle size and zeta potential) of lipoplexes vary with the different number of aliphatic chain. Subsequently, the different characterization of lipoplexes will affect the transfect process, such as the protection of pDNA or siRNA from nucleases, the endosomal escape and the pDNA and siRNA release from complex, which determine the final transfection efficiency conjointly.

### *In vitro* gene transfection

The transfection efficiency is one of the crucial parameters for active evaluation of non-viral gene delivery systems. Since the lipofection efficiency is a cell dependent process [55, 60], the *in vitro* transfection of these liposomes was evaluated using EGFP-C1 pDNA against six different cell lines including HEK293, Mat, PC-3, Hepg2, MCF-7 and HeLa cells. The commercially available gene transfection reagent, Lipofectamine 2000, was used as the positive control. Transfections with different N/P ratios were performed to find out the most effective formulations of lipid/pDNA complexes. As depicted in S3 Fig, Ara-DiC16MA liposome showed the highest transfection activity in all transfected cell lines compared with other three kinds of liposomes. At the same time, Ara-DiC18MA liposome has hardly transfection efficacy in all cell lines. It is possibly that Ara-DiC18MA liposome with a longer hydrophobic tails than other liposomes has greatly influence on process of formed liposome and lipoplex-membrane fusion [61]. Additionally, all the cationic lipids showed low gene transfection efficiency in the HeLa and MCF-7 cells. It appeared possibly that arabinose-based cationic lipoplexes has a bad cellular uptake and intracellular trafficking capability in this two cancer cell lines.

**Fig 4:**
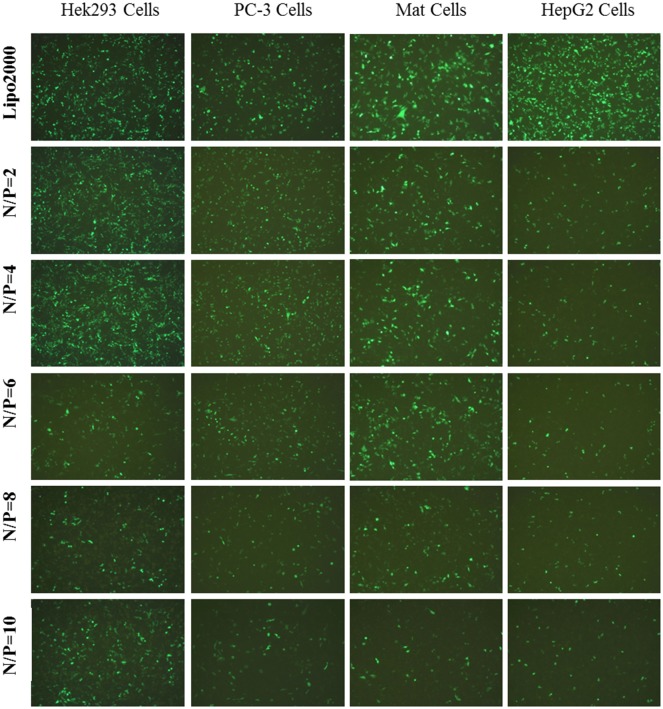
*In vitro* gene transfection. Fluorescents images (10×) of green fluorescent protein expression in Hek293, PC-3, Mat, HepG2 Cells using Ara-DiC16MA/pDNA complexes at N/P ratios of 2:1, 4:1, 6:1, 8:1. 10:1.

**Fig 5:**
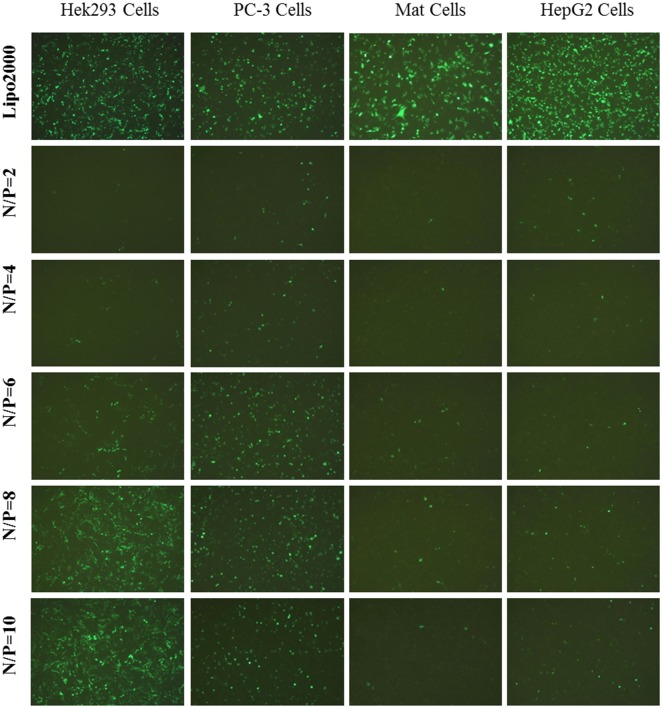
*In vitro* gene transfection. Transfection efficiency of Ara-DiC14MA/pDNA complexes in four kinds of cancer cells at different N/P ratios.

As shown in Fig 4, Ara-DiC16MA liposome has maximum transfection efficacy in HEK293, PC-3, Mat, HepG2 cell lines and the transfection efficacy decreased with the increase of the N/P ratio. Nevertheless, transfection efficacy of Ara-DiC14MA liposome increased with the increase of the N/P ratio in the cell lines mentioned above (Fig 5). The exceptional transfection efficacy of Ara-DiC14MA and Ara-DiC16MA liposomes perhaps due to the completely different capability of cellular uptake and release in transfected cells at different N/P ratios.

The transfection efficiencies of liposomes in HEK293 and PC-3 cells were also quantified by Cellometer K2 imager cytometer after transfection for 24 h. As shown in S4 Fig, with the N/P ratio of 2 and 4, the transfection efficacy of Ara-DiC16MA was quite equal to that of Lipofectamine 2000 in PC-3 cells and was slightly higher than that of Lipofectamine 2000 in HEK293 cells. Overall, the transfection efficacy of the four cationic liposomes except lipid Ara-DiC18MA increased with the increase of the hydrophobic chain length. The results showed that the hydrophobic chain length of cationic lipids and the N/P ratio of lipoplexes are essential factors to affect transfection efficiency.

### *In vitro* gene silencing

**Fig 6:**
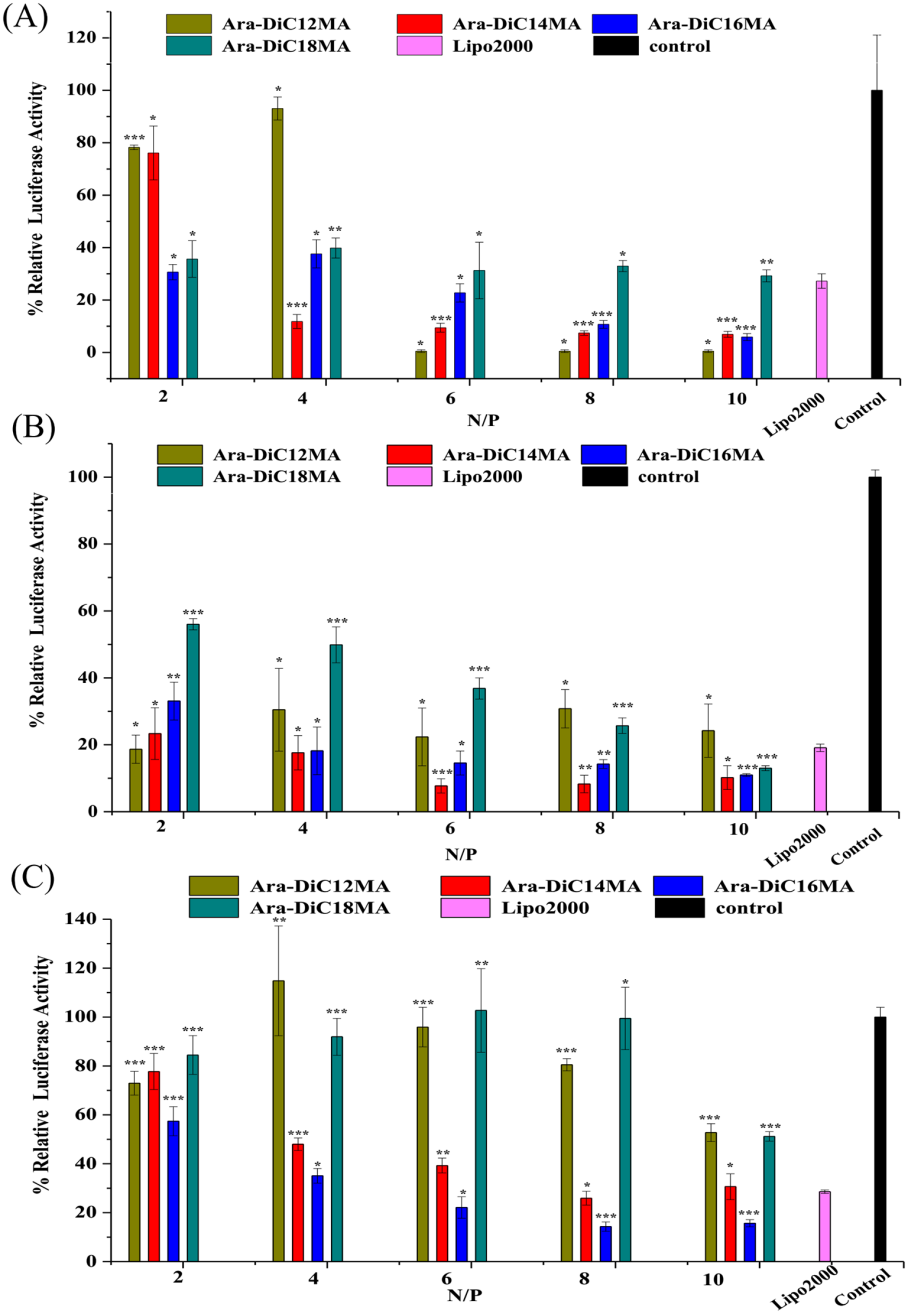
*In vitro* gene silencing. Silencing of a luciferase reporter protein in HEK293(A), PC-3(B), Mat(C) cells were conducted with the liposome/siRNA complexes (Ara-DiC12MA, Ara-DiC14MA, Ara-DiC16MA, Ara-DiC18MA). The mean luciferase activity was represented by relative light unit and was calculated from three different measurements. Statistical differences from the Lipo2000 are labelled * P < 0.05, ** P < 0.005 and *** P< 0.001.

According to the results of EGFP-C1 pDNA transfection, HEK293, PC-3 and Mat cells were also selected to investigate the efficiency of siRNA delivery *in vitro* by using luciferase knockdown assay. The cells were treated with Liposome/siRNA and then loaded with dual-luciferase report gene mediated by Lipofectamine 2000. The siRNA can specifically and efficiently inhibit the expression of firefly luciferase while renal luciferase activity remains intact. By measuring the ratio of firefly/renal, we can estimate RNA interference (RNAi) efficiency. Meanwhile, Lipofectamine2000/siRNA and phosphate-buffered saline were set as positive and negative control respectively. In vitro gene silencing of each cationic carrier at N/P ratios of 2, 4, 6, 8 and 10 were tested to determine the optimal N/P ratio with the best RNAi efficiency. The gene silencing activity was reported as % relative luciferase activity as shown in Fig 6. Higher RNAi activity was observed in HEK293 cells incubated with cationic lipids of Ara-DiC14MA, Ara-DiC16MA and Ara-DiC18MA at various N/P charge ratios. The lower relative luciferase activity was also observed in cells transfected with lipid Ara-DiC12MA at N/P ratios of 6:1, 8:1 and 10:1 than at N/P ratios of 2:1 and 4:1. Remarkably, 0.5% luciferase activity is not representative of silencing activity of lipid Ara-DiC12MA (Fig 6A), because of most cells died with lipolexes at N/P of 6:1, 8:1 and 10:1. The siRNA-mediated gene knockdown of the cationic lipids was further determined in PC-3 cell, as shown in Fig 6B. The similar results were also found that Ara-DiC14MA and Ara-DiC16MA lipids achieved greater than 80–90% knockdown efficiency at all N/P ratios except 2:1.

The lipids/siRNA complexes of Ara-DiC14MA and Ara-DiC16MA at N/P ratios of 2:1, 4:1, 6:1, 8:1, 10:1 showed gradually increasing trend of RNAi activity compared with the other two lipids/siRNA complexes in Mat cells. Apparently, Ara-DiC16MA showed higher RNAi efficiencies than the Lipo2000 at N/P ratios of 6:1, 8:1, 10:1. Nevertheless, compared with the negative control, Ara-DiC12MA and Ara-DiC18MA have almost no RNAi activity at N/P ratios of 2:1, 4:1, 6:1, 8:1 except 10:1 (Fig 6C). In a word, it is reasonable to believe that lipid Ara-DiC14MA and Ara-DiC16MA might be potential lipid carriers for efficient siRNA encapsulation and delivery.

### Cellular uptake of lipid/pDNA lipoplexes

**Fig 7:**
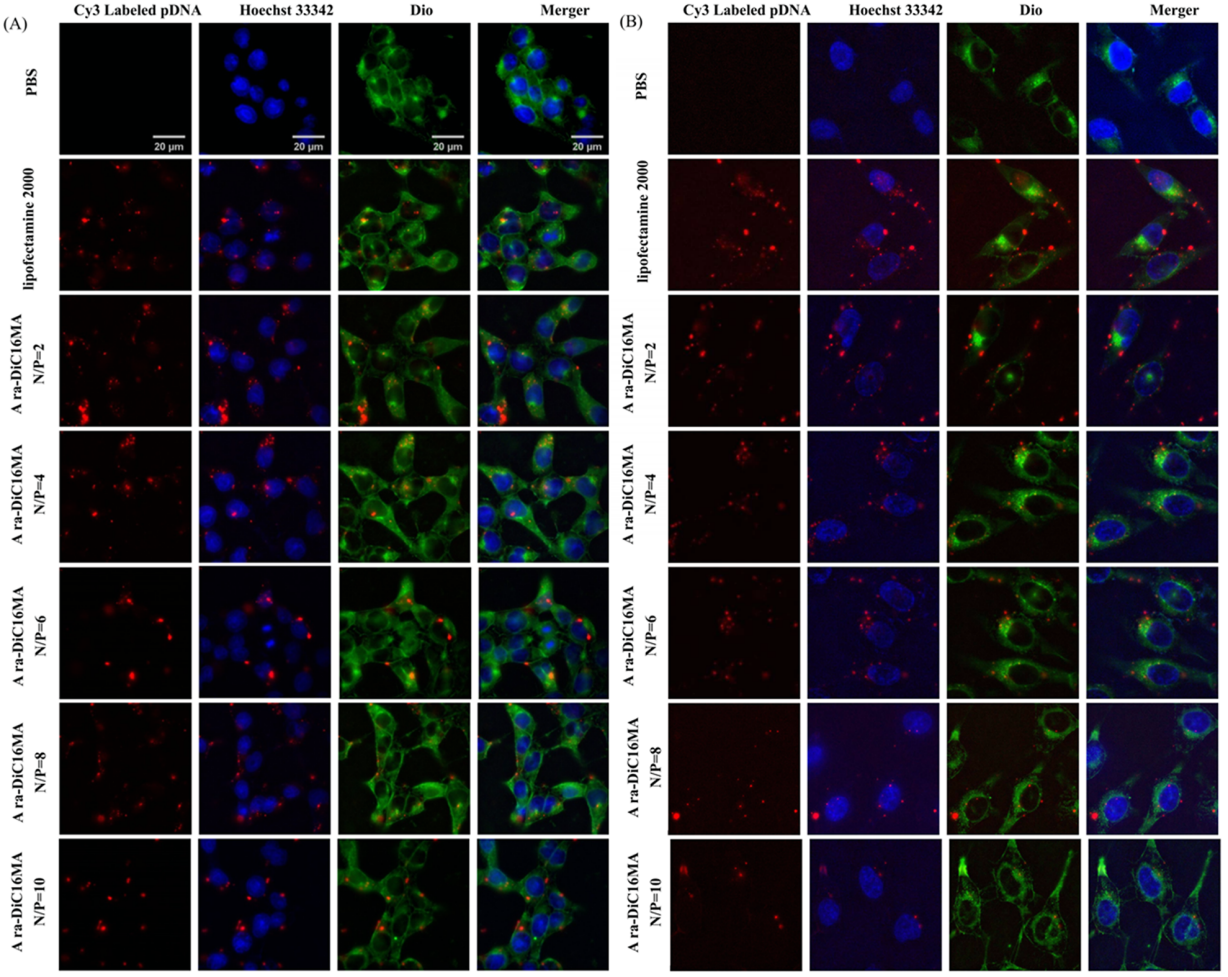
Cellular uptake of lipid/pDNA lipoplexes. Fluorescence microscopic images (100×) of cellular uptake of Ara-DiC16MA Lipids/pDNA complexes at the N/P ratio of 2:1, 4:1, 6:1, 8:1, 10:1 in HEK293(A) and Mat (B) after 4 h of gene transfection. Bar: 20 μm. (green: Dio used to label cytomembrane, red: Cy3-labeled pDNA, blue: Hochest 33342 stained cell nuclei).

To ascertain whether cationic liposomes can provide effective cellular uptake of pDNA into HEK293, PC-3, Mat and HeLa cells. Dio was used to stain the cytomembrane (green), Hochest 33342 was used to label the nuclei (blue), Cy3-labeled pDNA (red) was used to trace the lipoplexes. Lipofectamine2000 and PBS were set as positive and negative control, respectively. As showed in Fig 7 and Supplementary S5 Fig, qualitative cellular uptake was clearly apparent that the lipid-associated red fluorescent pDNA is distributed in part within the cell cytoplasm (green) and in part on the periphery of cell nuclei (blue). The higher uptake of lipoplexes labeled by Cy3-labeled pDNA was found in HEK293, PC-3 and Mat cells than in HeLa cell (Supplementary S3F and S5C Figs), which is consistent with the results of transfection efficiency. Overall, these researches suggest that a higher cellular uptake capability of the liposomes/pDNA complexes is expected to enhance gene delivery and expression.

### *In vitro* cytotoxicity

**Fig 8:**
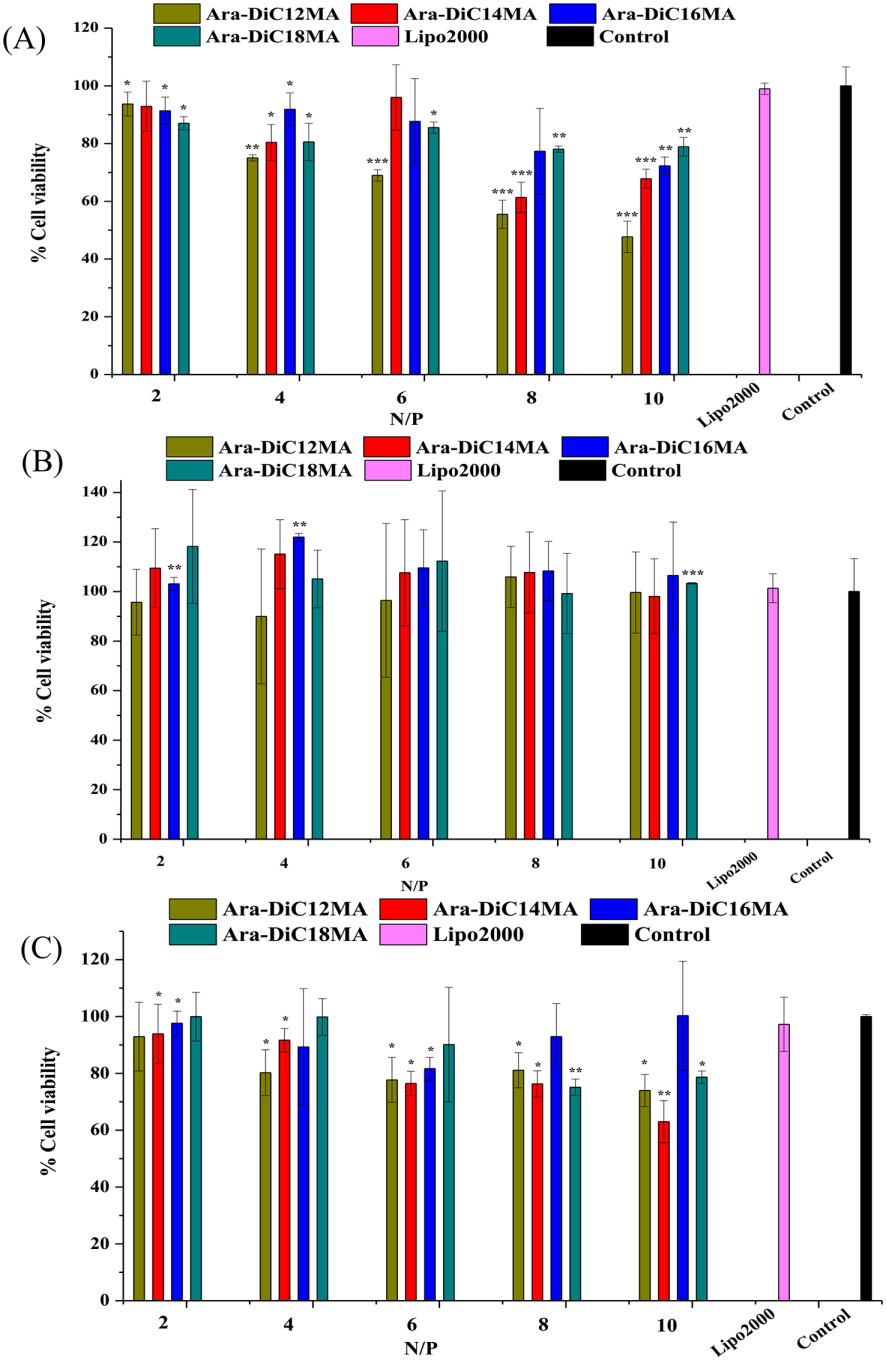
*In vitro* cytotoxicity. The cell toxicity of cationic lipoplexes (Ara-DiC12MA, Ara-DiC14MA, Ara-DiC16MA, Ara-DiC18MA) in Hek293 (A), Mat (B) and PC-3 cells (C). The cytotoxicity of Lipofectamine2000/pDNA and untreated cells were detected as the positive and normalized control, respectively. The mean cell viability was calculated from three different measurements. Statistical differences from the Lipo2000 are labelled * P < 0.05, ** P < 0.005 and *** P< 0.001.

In general, the clinical success of cationic liposomes carriers for gene delivery is characterized by maximum efficiency and minimum toxicity [62]. The *in vitro* cytotoxicity of cationic lipids/pDNA complexes were established on all transfected cell lines using MTT assay. In viable cells, the solution was formed by dissolution of insoluble purple formazan in DMSO, which has absorbance at 490 nm. As shown in Figs 8 and S6, the four cationic lipoplexes showed minimal cytotoxicity (viability more than 80%) in HEK93, Mat, PC-3 and HepG2 cells except Ara-DiC12MA lipoplexes. Nonetheless, Ara-DiC12MA and Ara-DiC14MA liposomes induce largely cell death in MCF-7 (S6B Fig) and HeLa cells (S6C Fig). The lower cytotoxicity of the lipoplexes showed the L-arabinose-based glycolipids have good biocompatibility, and also might be one of the reasons for its higher transfection efficacies, gene silencing and cellular uptake ability, especially for lipid Ara-DiC16MA.

## Conclusions

In summary, as candidates for non-viral gene vectors, a new kind of L-arabinose based cationic lipids bearing different hydrophobic alkane chain were successfully synthesized. Gel electrophoresis assay, AFM images and dynamic light scattering experiments demonstrate that all the liposomes could efficiently bind and compact pDNA and siRNA into nanoparticles with proper size and zeta potential. The gene transfection study showed that lipid Ara-DiC16MA has maximum transfection efficacy in HEK293, PC-3 and Mat cells compared with other three kinds of liposomes, which maintains low cytotoxicity and better cellular uptake capability *in vitro*. The luciferase knockdown experiments of Ara-DiC16MA and Ara-DiC14MA liposomes demonstrated the higher RNAi activity for siRNA delivery than Lipofectamine 2000 at high N/P ratio. We believed that L-arabinose-based cationic liposomes with differences hydrophobic alkane chain may be a key factor to ensure highly efficient transfection *in vitro*. Additionally, these works strengthen the validity of the carbohydrates based cationic lipids as effective non-viral gene vectors for gene delivery.

## Supporting information

S1 Fig Particle size, zeta potential and morphology measurements. (A) Mean particle size and (B) zeta potential of the liposomes/siRNA complexes at different N/P ratios. AFM morphologies images of Ara-DiC14MA/siRNA complex at N/P ratio of 4(C) and 6 (D) and Ara-DiC16MA /siRNA complex at N/P ratio of 4(E) and 6 (F). Each value represents the mean ± standard deviation of three measurements.
(DOCX)

S2 Fig Determination of siRNA affinity by agarose gel electrophoresis. Gel electrophoresis assay of Ara-DiC12MA/siRNA complexes (A), Ara-DiC14MA/ siRNA complexes (B), Ara-DiC16MA/siRNA complexes (C), Ara-DiC18MA/siRNA complexes (D) at five N/P ratios of 0.3:1, 05:1, 1:1, 2:1 and 3:1.
(DOCX)

S3 Fig *In vitro* gene transfection. Transfection efficiency of lipid/pDNA complexes in HEK293(A), Mat(B), PC-3(C), Hepg2(D), MCF-7(E) and HeLa(F) cells at different N/P ratios of 2:1, 4:1, 6:1, 8:1, 10:1.
(DOCX)

S4 Fig Quantitative analysis of gene expression. Transfection efficiency of Ara-DiC16MA /pDNA complexes in PC-3 and HEK293 cells at different N/P ratios (2:1–10:1). Lipo2000 (2 μL) was used as the positive control. Each value represents the mean ± standard deviation of three measurements. Statistical differences from the Lipo2000 are labelled * P < 0.05, ** P < 0.005 and *** P< 0.001.
(DOCX)

S5 Fig Cellular uptake of lipid/pDNA lipoplexes. Fluorescence microscopic images (100×) of cellular uptake in PC-3 cell (A, Ara-DiC16MA/pDNA complexes), HEK293 cell (B, Ara-DiC14MA/pDNA complexes) and HeLa (C, Ara-DiC16MA/pDNA complexes) at the N/P ratio of 2:1, 4:1, 6:1, 8:1, 10:1 after 4 h of gene transfection (green: Dio used to label cytomembrane, red: Cy3-labeled pDNA, blue: Hochest 33342 stained cell nuclei).
(DOCX)

S6 Fig *In vitro* cytotoxicity. The cell toxicity of HepG2 (A), MCF-7(B) and HeLa(C) treated with cationic lipoplexes (Ara-DiC12MA, Ara-DiC14MA, Ara-DiC16MA, Ara-DiC18MA) at different N/P ratios. The mean cell viability was calculated from three different measurements. Statistical differences from the Lipo2000 are labelled * P < 0.05, ** P < 0.005 and *** P< 0.001.
(DOCX)

S1 Table Mean particle size and zeta potential of the lipid/pDNA complexes at different N/P ratios. (DOCX)

S2 Table Mean particle size and zeta potential of the lipid/siRNA complexes at different N/P ratios. (DOCX)

S1 File The procedure for synthesis of lipids 9a-9d. (DOCX)

S2 File The NMR spectrum of lipids 9a-9d and partial intermediates. (DOCX)
