# Supplementary material for: Synthesis and evaluation of L-arabinose-based cationic glycolipids as effective vectors for pDNA and siRNA in vitro
Source: PLoS One. 2017 Jul 3;12(7):e0180276. doi: 10.1371/journal.pone.0180276 (PMC5495346; doi:10.1371/journal.pone.0180276)
Supplement: S1 Fig — (A) Mean particle size and (B) zeta potential of the liposomes/siRNA complexes at different N/P ratios. AFM morphologies images of Ara-DiC14MA/siRNA complex at N/P ratio of 4(C) and 6 (D) and Ara-DiC16MA /siRNA complex at N/P ratio of 4(E) and 6 (F). Each value represents the mean ± standard deviation of three measurements. (DOCX) [file pone.0180276.s001.docx]

(D)

(C)

(B)

(A)


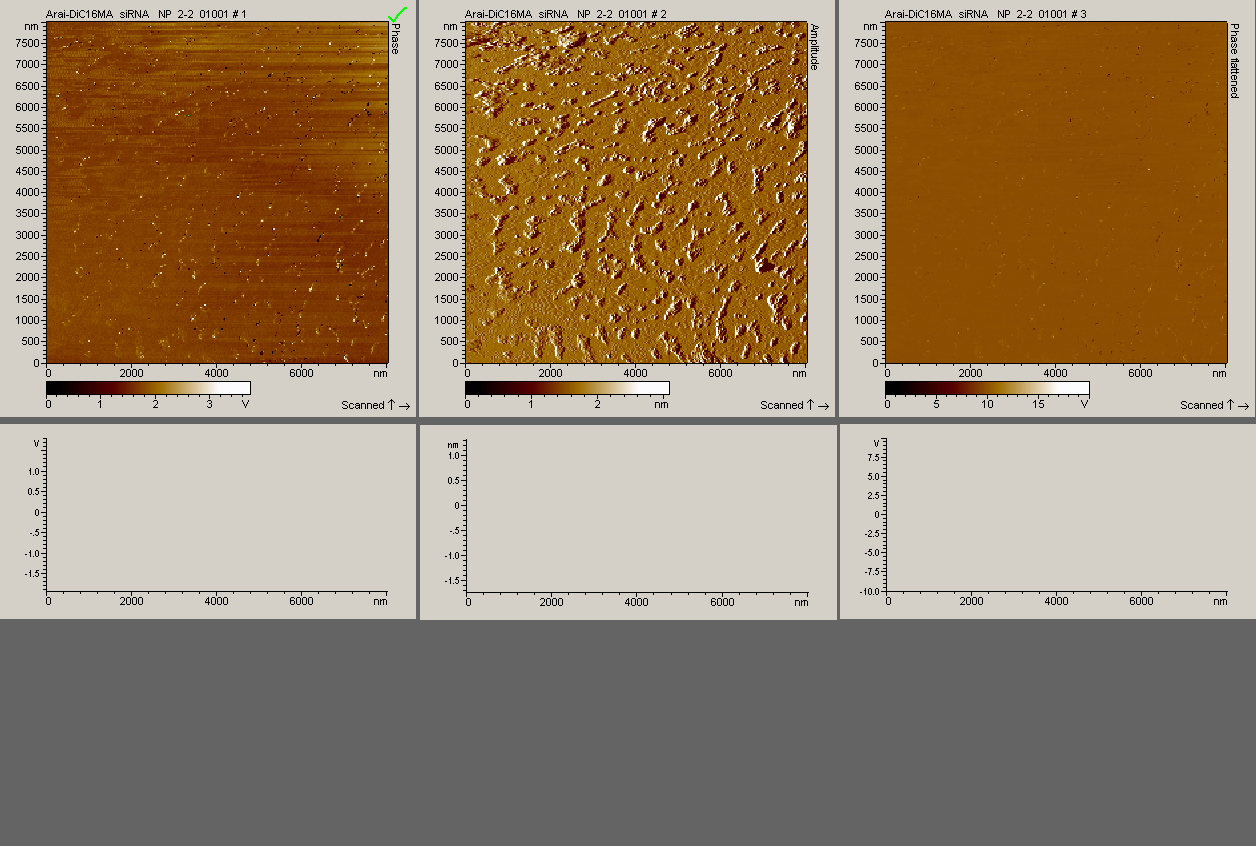
 **
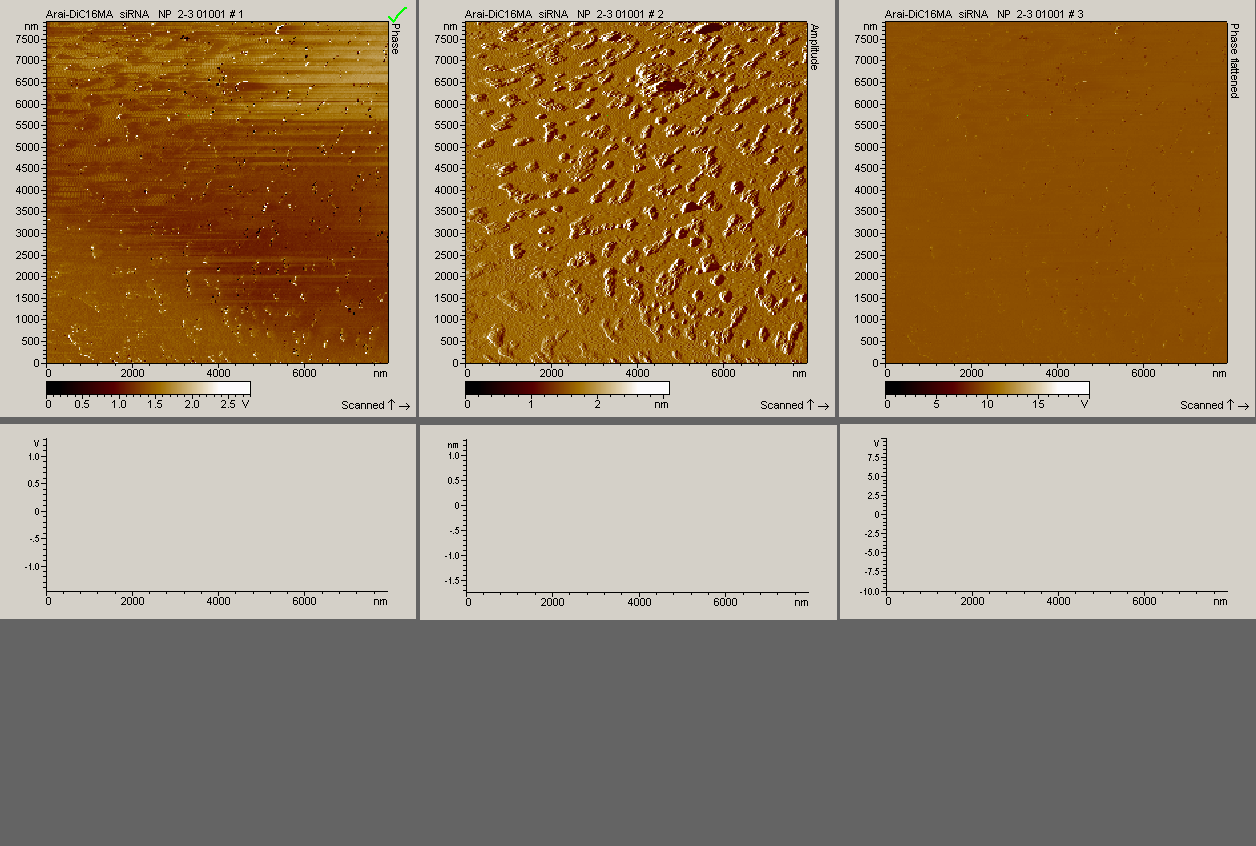
**

(F)

(E)

**
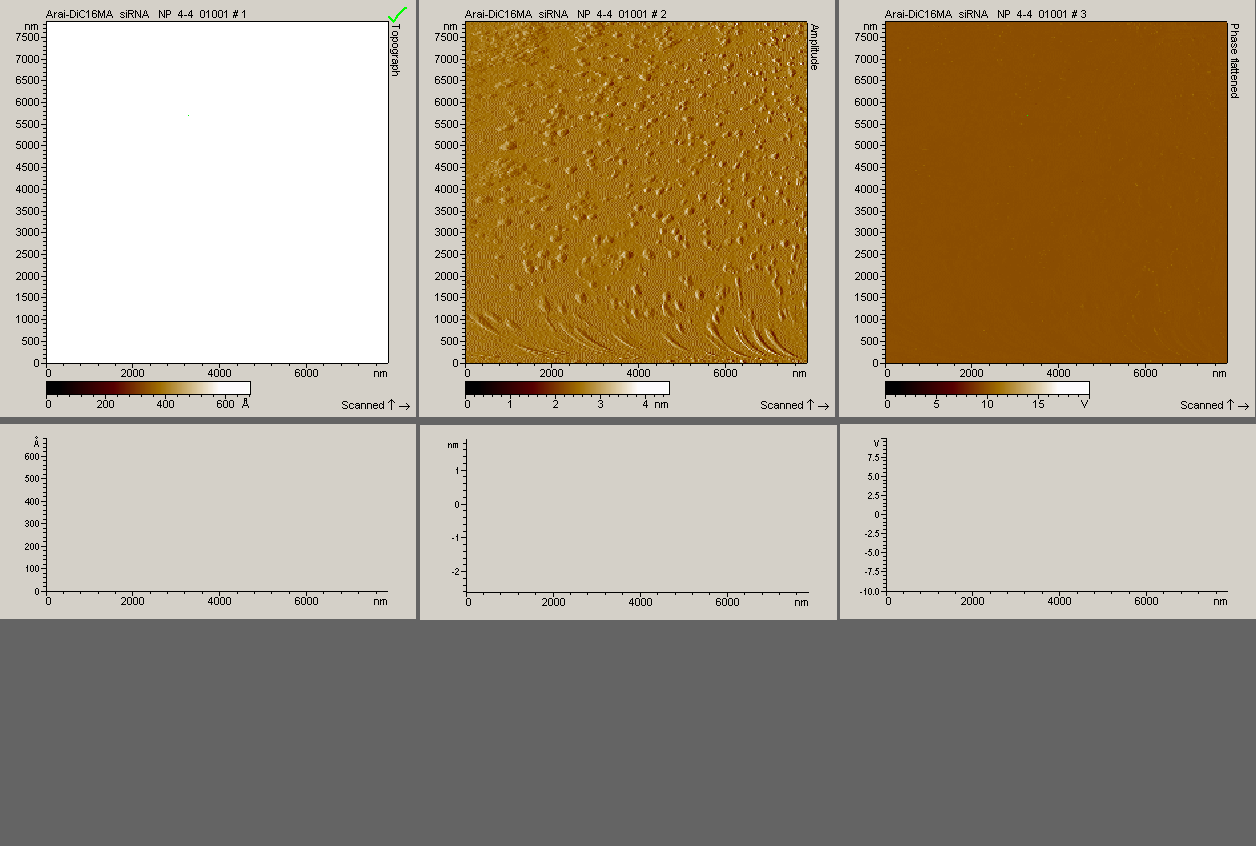

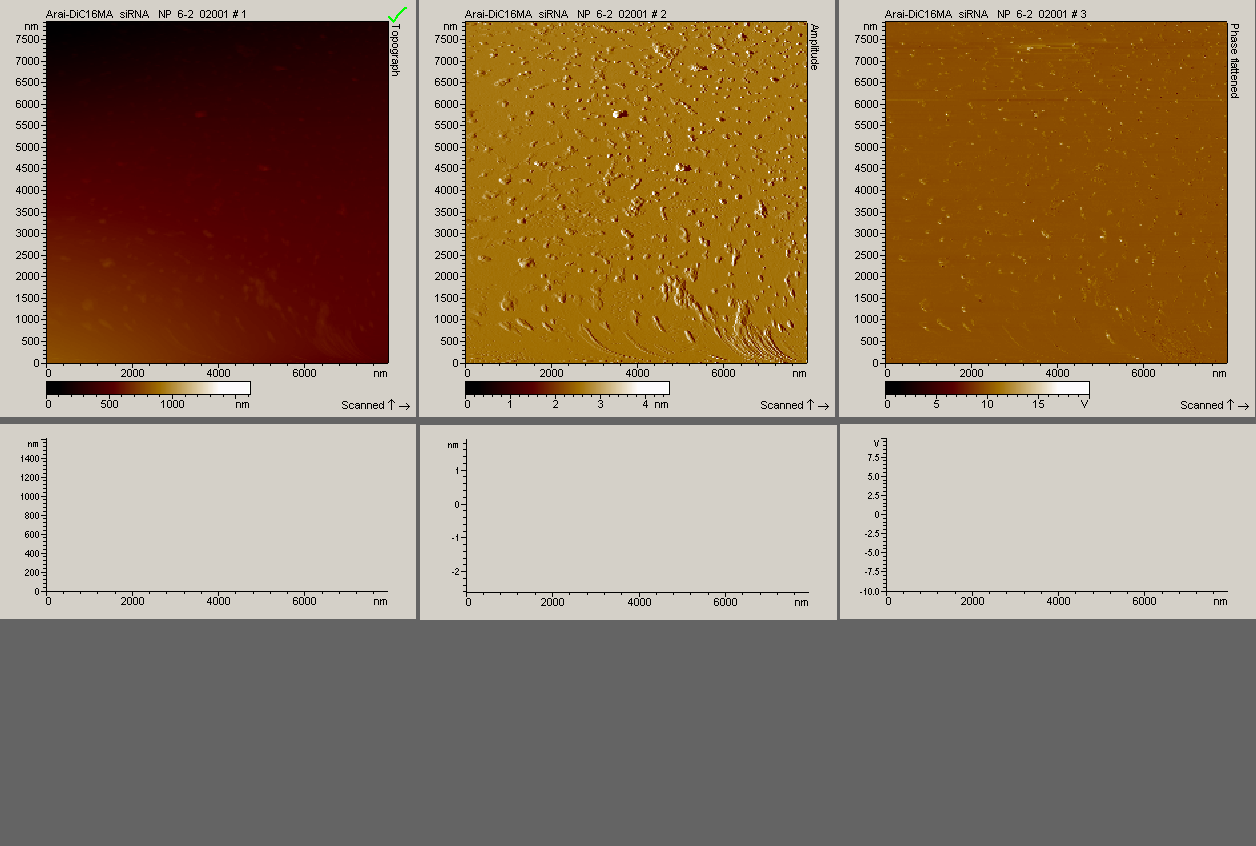
**

**S1 Fig. Particle size, zeta potential and morphology measurements.** (A) Mean particle size and (B) zeta potential of the liposomes/siRNA complexes at different N/P ratios. AFM morphologies images of Ara-DiC14MA/siRNA complex at N/P ratio of 4 (C) and 6 (D) and Ara-DiC16MA /siRNA complex at N/P ratio of 4 (E) and 6 (F). Each value represents the mean ± standard deviation of three measurements.
