# Supplementary material for: Synthesis and evaluation of L-arabinose-based cationic glycolipids as effective vectors for pDNA and siRNA in vitro
Source: PLoS One. 2017 Jul 3;12(7):e0180276. doi: 10.1371/journal.pone.0180276 (PMC5495346; doi:10.1371/journal.pone.0180276)
Supplement: S2 Fig — Gel electrophoresis assay of Ara-DiC12MA/siRNA complexes (A), Ara-DiC14MA/ siRNA complexes (B), Ara-DiC16MA/siRNA complexes (C), Ara-DiC18MA/siRNA complexes (D) at five N/P ratios of 0.3:1, 05:1, 1:1, 2:1 and 3:1. (DOCX) [file pone.0180276.s002.docx]

**(A )** siRNA 0.3 0.5 1 2 3 (**B)** siRNA 0.3 0.5 1 2 3


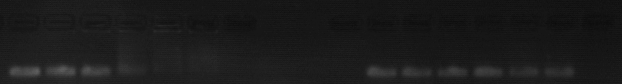

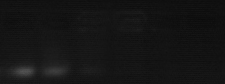


**(C)** siRNA 0.3 0.5 1 2 3 (**D)** siRNA 0.3 0.5 1 2 3


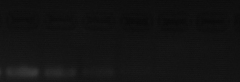

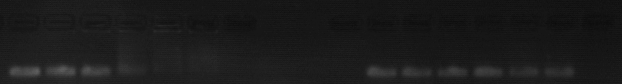


**S2 Fig.** **Determination of siRNA affinity by agarose gel electrophoresis.** Gel electrophoresis assay of Ara-DiC12MA/siRNA complexes (A), Ara-DiC14MA/ siRNA complexes (B), Ara-DiC16MA/siRNA complexes (C), Ara-DiC18MA/siRNA complexes (D) at five N/P ratios of 0.3:1, 05:1, 1:1, 2:1 and 3:1.
