# Supplementary material for: Synthesis and evaluation of L-arabinose-based cationic glycolipids as effective vectors for pDNA and siRNA in vitro
Source: PLoS One. 2017 Jul 3;12(7):e0180276. doi: 10.1371/journal.pone.0180276 (PMC5495346; doi:10.1371/journal.pone.0180276)
Supplement: S3 Fig — Transfection efficiency of lipid/pDNA complexes in HEK293(A), Mat(B), PC-3(C), Hepg2(D), MCF-7(E) and HeLa(F) cells at different N/P ratios of 2:1, 4:1, 6:1, 8:1, 10:1. (DOCX) [file pone.0180276.s003.docx]

(**A**) N/P=2 N/P=4 N/P=6 N/P=8 N/P=10 Lipo2000

Lipid **9d** Lipid **9c** Lipid **9b** Lipid **9a**


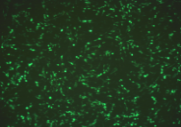

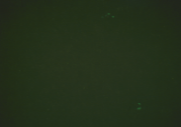

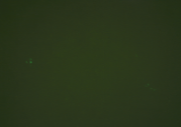

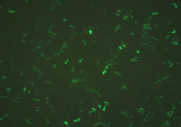

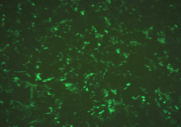

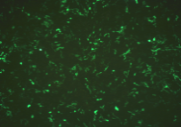

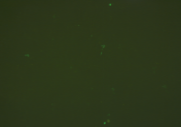

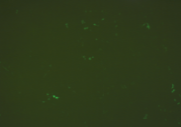

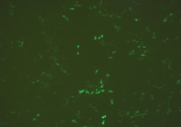

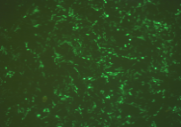

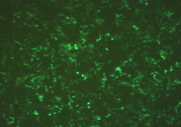

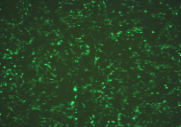

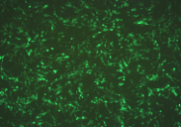

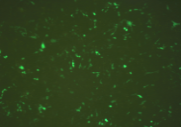

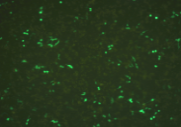

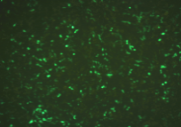

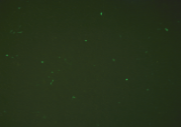

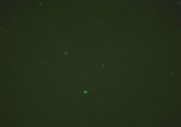

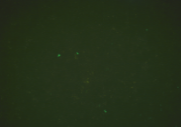

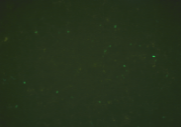

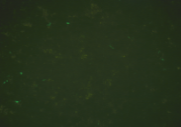


(**B**) N/P=2 N/P=4 N/P=6 N/P=8 N/P=10 Lipo2000

Lipid **9d** Lipid **9c** Lipid **9b** Lipid **9a**


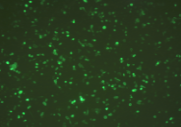

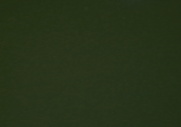

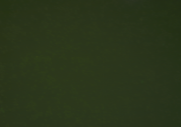

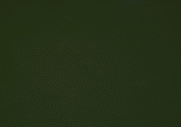

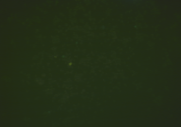

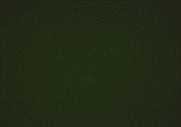

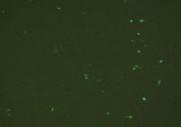

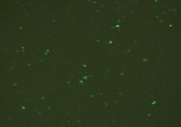

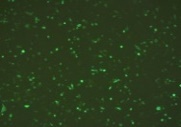

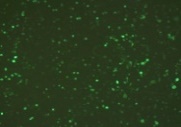

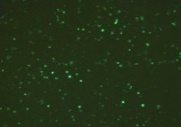

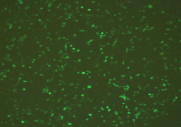

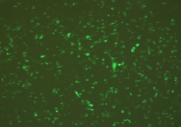

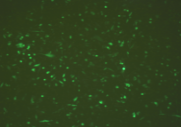

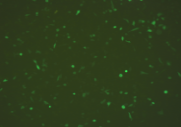

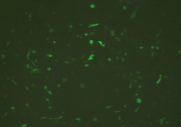

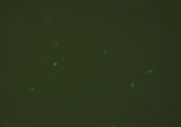

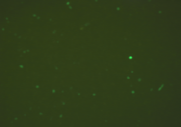

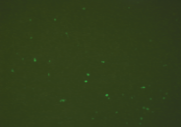

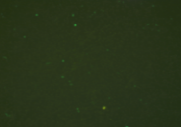

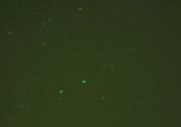


(**C**) N/P=2 N/P=4 N/P=6 N/P=8 N/P=10 Lipo2000

Lipid **9d** Lipid **9c** Lipid **9b** Lipid **9a**


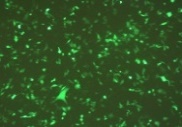

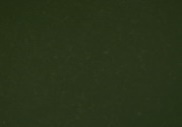

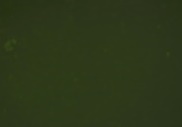

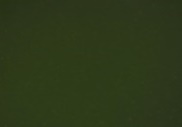

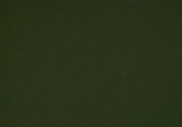

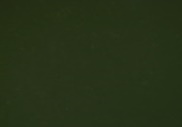

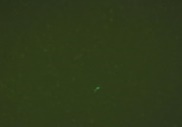

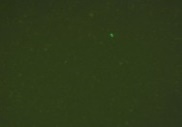

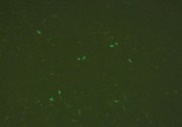

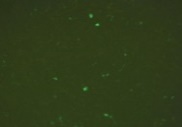

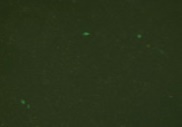

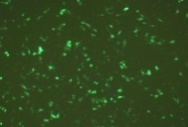

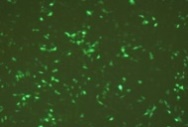

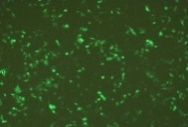

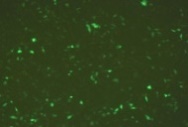

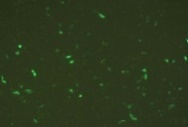

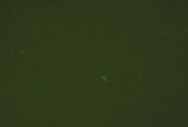

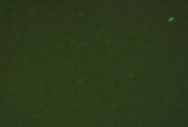

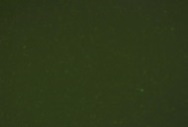

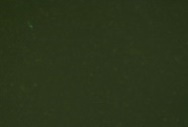

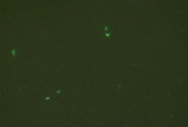


(**D**) N/P=2 N/P=4 N/P=6 N/P=8 N/P=10 Lipo2000

Lipid **9d** Lipid **9c** Lipid **9b** Lipid **9a**


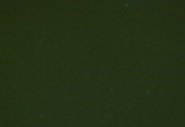

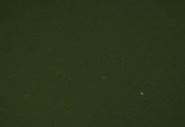

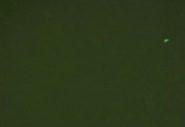

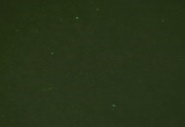

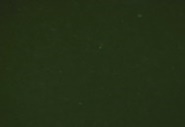

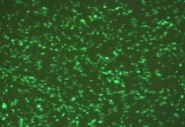

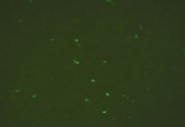

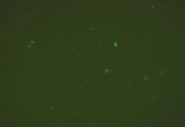

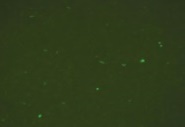

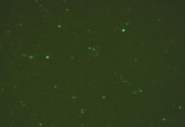

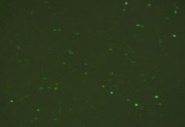

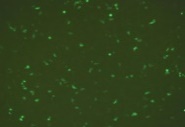

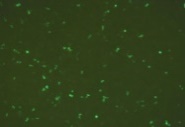

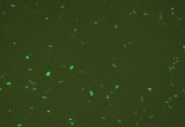

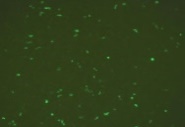

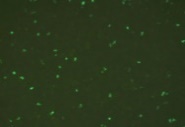

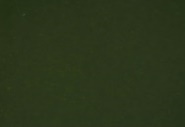

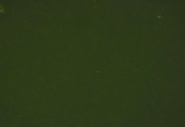

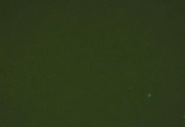

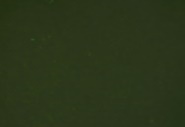

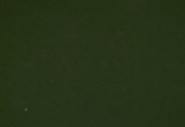


(**E**) N/P=2 N/P=4 N/P=6 N/P=8 N/P=10 Lipo2000

Lipid **9d** Lipid **9c** Lipid **9b** Lipid **9a**


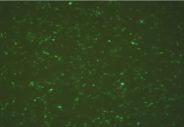

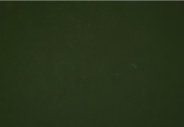

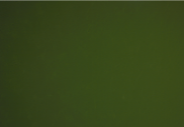

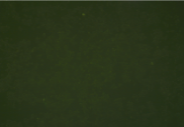

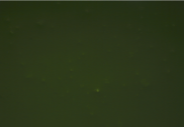

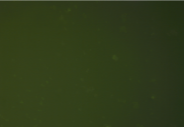

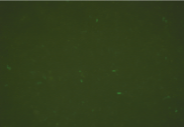

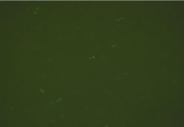

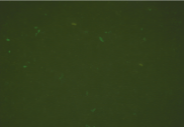

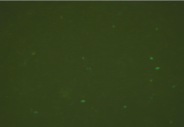

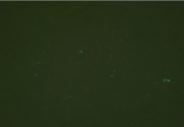

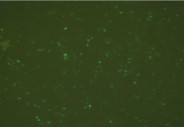

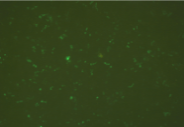

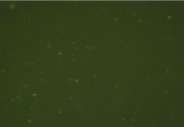

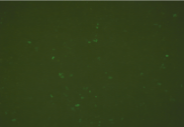

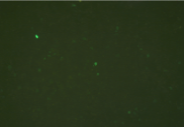


(**F**) N/P=2 N/P=4 N/P=6 N/P=8 N/P=10 Lipo2000

Lipid **9d** Lipid **9c** Lipid **9b** Lipid **9a**

**S3 Fig. *In vitro* gene transfection.** Transfection efficiency of lipid/pDNA complexes in HEK293(A), Mat(B), PC-3(C), Hepg2(D), MCF-7(E) and HeLa(F) cells at different N/P ratios of 2:1, 4:1, 6:1, 8:1, 10:1.
