# Supplementary material for: Synthesis and evaluation of L-arabinose-based cationic glycolipids as effective vectors for pDNA and siRNA in vitro
Source: PLoS One. 2017 Jul 3;12(7):e0180276. doi: 10.1371/journal.pone.0180276 (PMC5495346; doi:10.1371/journal.pone.0180276)
Supplement: S4 Fig — Transfection efficiency of Ara-DiC16MA /pDNA complexes in PC-3 and HEK293 cells at different N/P ratios (2:1–10:1). Lipo2000 (2 μL) was used as the positive control. Each value represents the mean ± standard deviation of three measurements. Statistical differences from the Lipo2000 are labelled * P < 0.05, ** P < 0.005 and *** P< 0.001. (DOCX) [file pone.0180276.s004.docx]

**S4 Fig. Quantitative analysis of gene expression**. Transfection efficiency of Ara-DiC16MA /pDNA complexes in PC-3 and HEK293 cells at different N/P ratios (2:1-10:1). Lipo2000 (2 μL) was used as the positive control. Each value represents the mean ± standard deviation of three measurements. Statistical differences from the Lipo2000 are labelled * P < 0.05, ** P < 0.005 and *** P< 0.001.
