# Supplementary material for: Synthesis and evaluation of L-arabinose-based cationic glycolipids as effective vectors for pDNA and siRNA in vitro
Source: PLoS One. 2017 Jul 3;12(7):e0180276. doi: 10.1371/journal.pone.0180276 (PMC5495346; doi:10.1371/journal.pone.0180276)
Supplement: S5 Fig — Fluorescence microscopic images (100×) of cellular uptake in PC-3 cell (A, Ara-DiC16MA/pDNA complexes), HEK293 cell (B, Ara-DiC14MA/pDNA complexes) and HeLa (C, Ara-DiC16MA/pDNA complexes) at the N/P ratio of 2:1, 4:1, 6:1, 8:1, 10:1 after 4 h of gene transfection (green: Dio used to label cytomembrane, red: Cy3-labeled pDNA, blue: Hochest 33342 stained cell nuclei). (DOCX) [file pone.0180276.s005.docx]

(A)

**Cy3 Labeled DNA Hoechst 33342 Dio Merger**


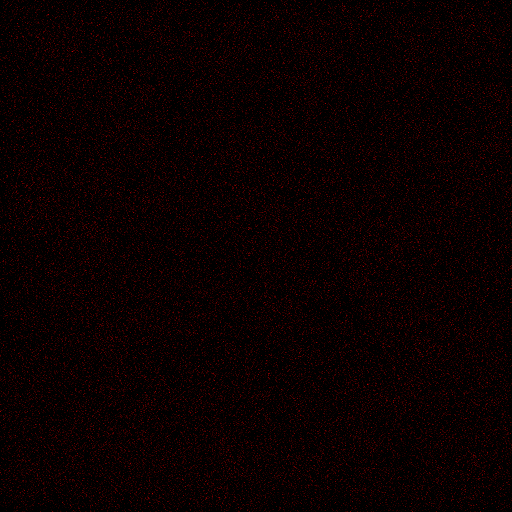

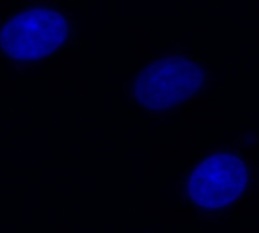

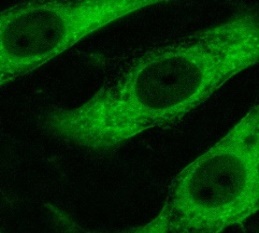

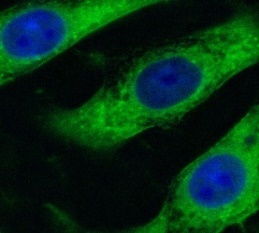


**PBS**

**lipofectamine 2000**


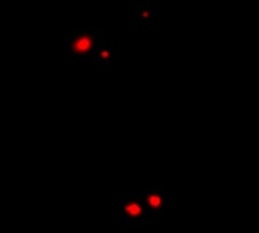

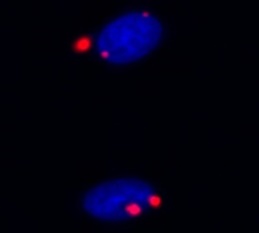

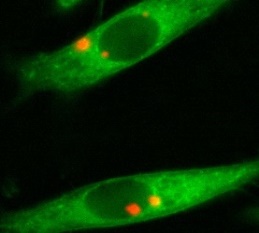

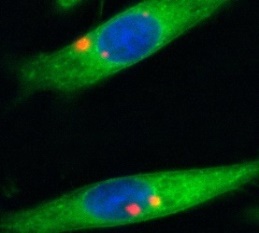


**A ra-DiC16MA**

**N/P=2**


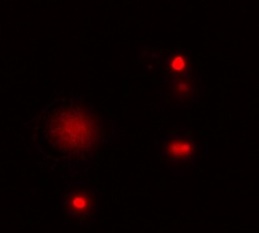

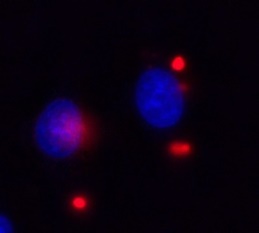

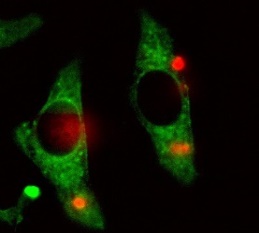

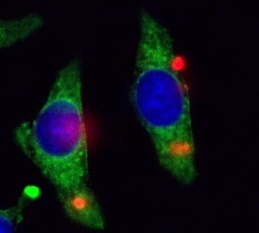


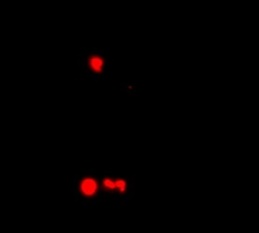

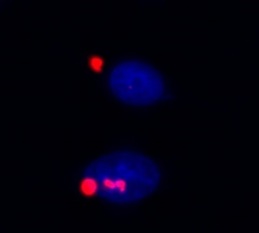

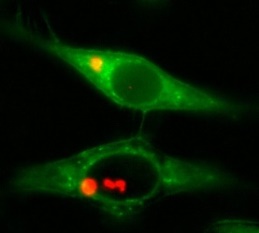

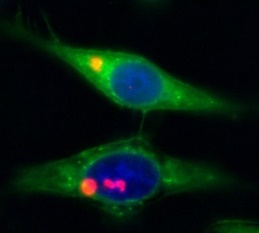


**A ra-DiC16MA**

**N/P=6**

**A ra-DiC16MA**

**N/P=4**


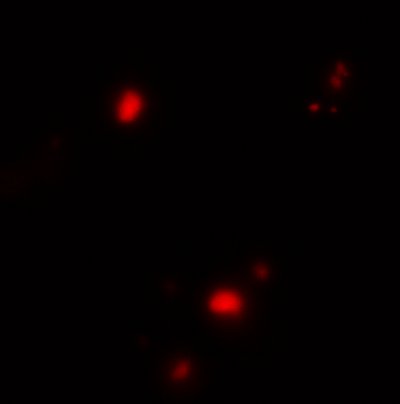

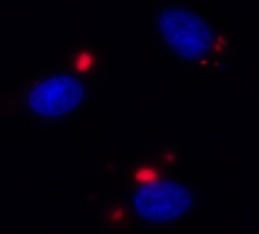

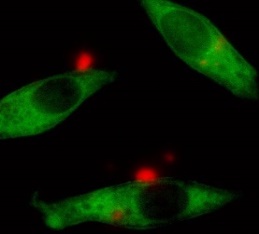

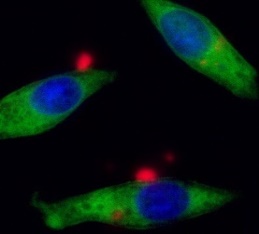


**A ra-DiC16MA**

**N/P=8**


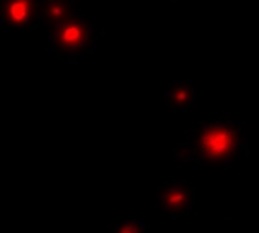

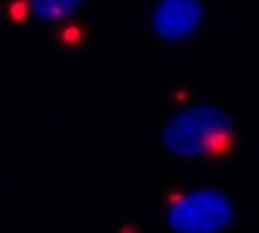

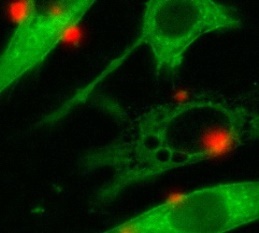

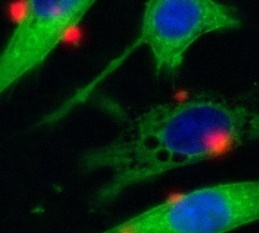


**A ra-DiC16MA**

**N/P=10**


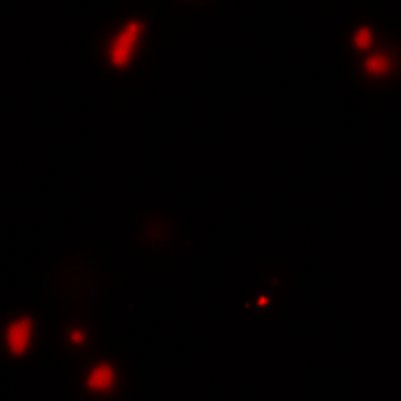

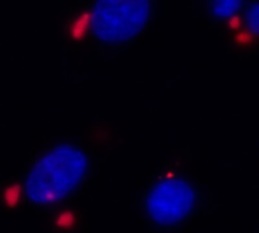

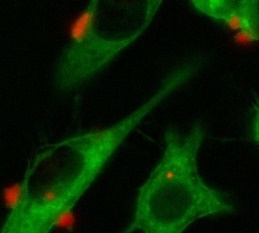

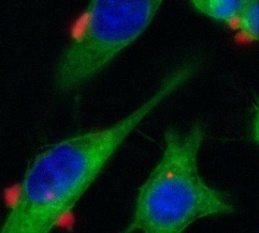


(B)

**Cy3 Labeled DNA Hoechst 33342 Dio Merger**


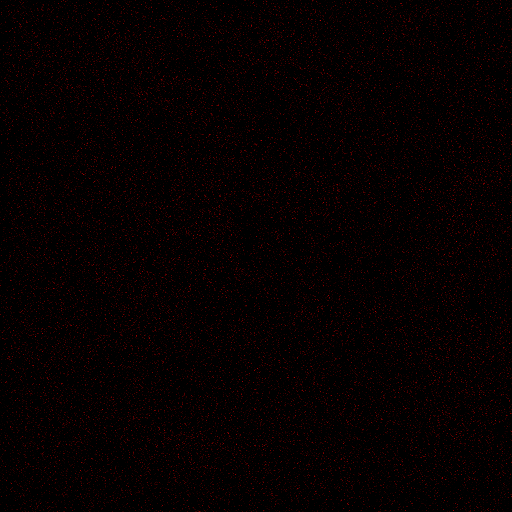

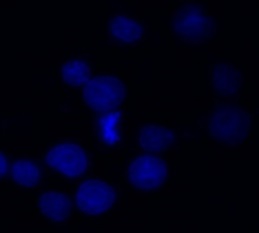

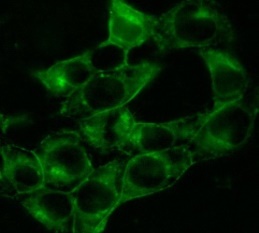

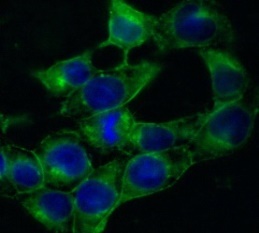


**PBS**

**lipofectamine 2000**


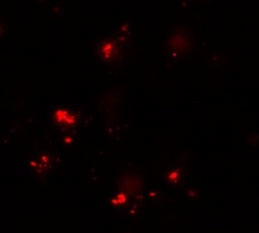

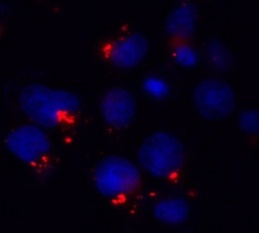

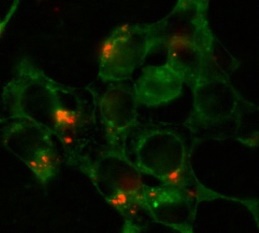

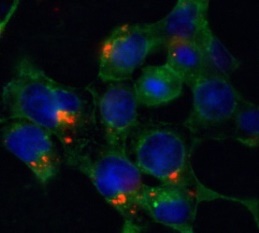


**A ra-DiC14MA**

**N/P=2**


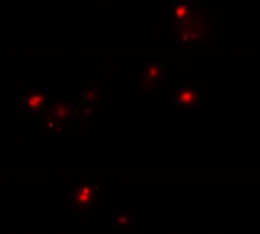

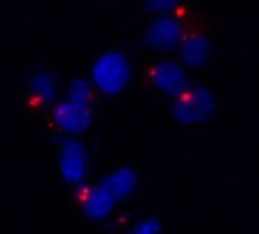

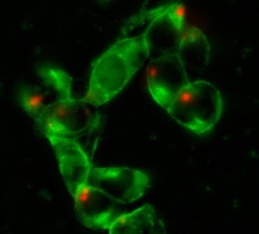

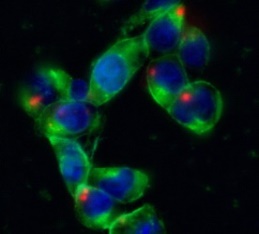


**A ra-DiC14MA**

**N/P=4**

**
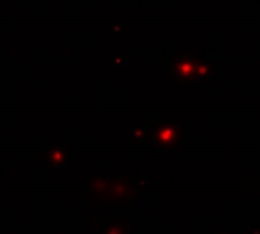

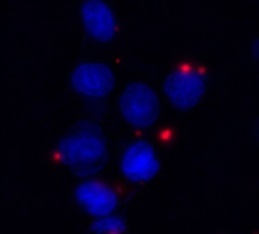
**  **
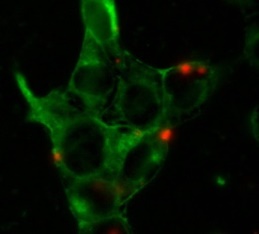

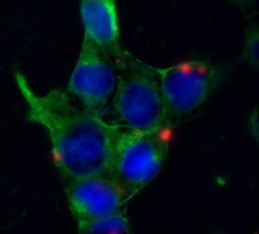
**

**A ra-DiC14MA**

**N/P=6**

**
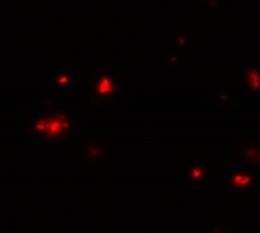

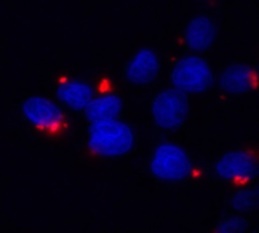

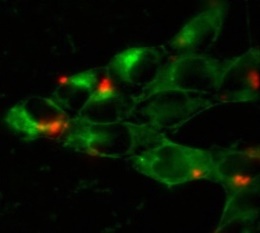

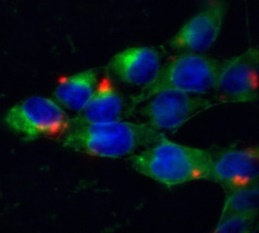
**

**A ra-DiC14MA**

**N/P=8**


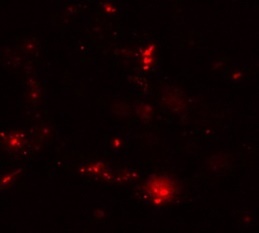

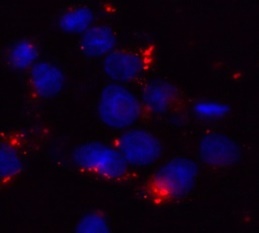

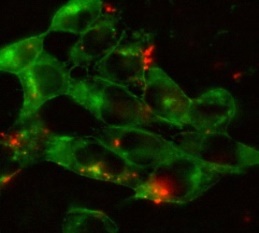

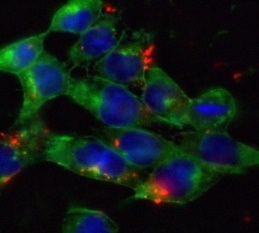


**A ra-DiC14MA**

**N/P=10**


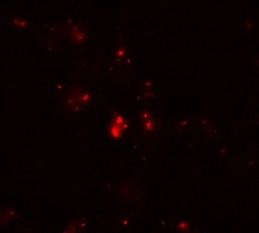

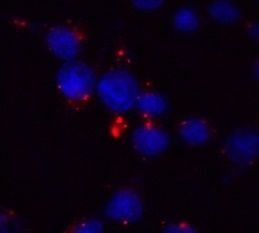

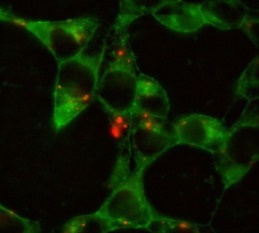

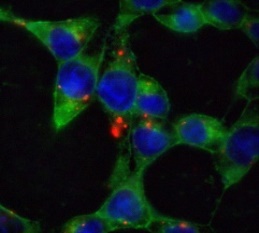


(C)

**Cy3 Labeled DNA Hoechst 33342 Dio Merger**


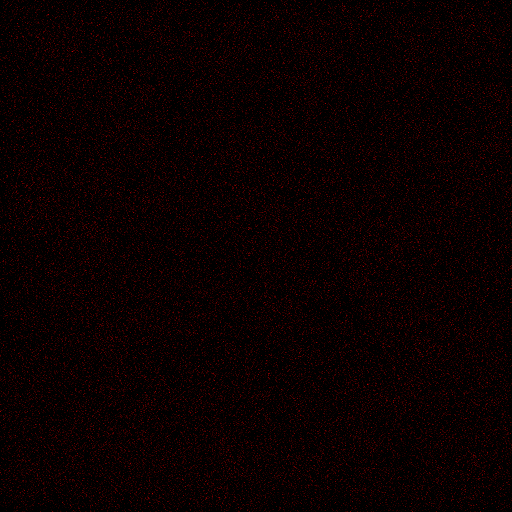

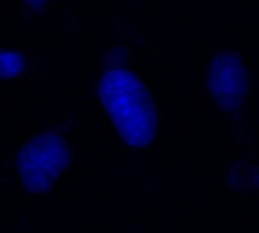

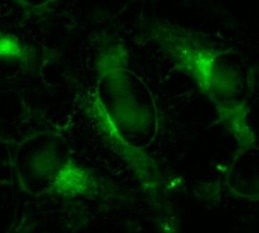

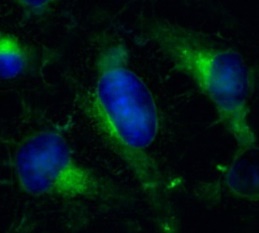


**lipofectamine 2000**

**PBS**


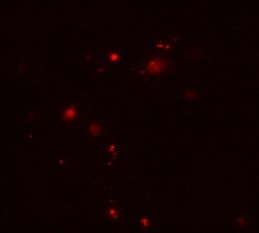

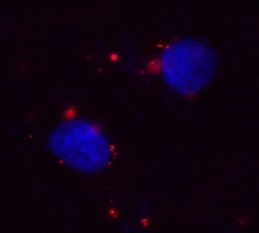

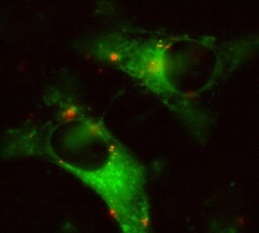

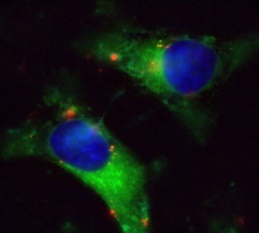


**A ra-DiC16MA**

**N/P=2**


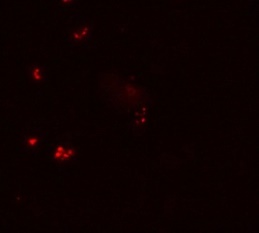

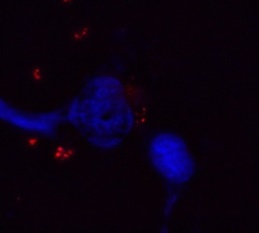

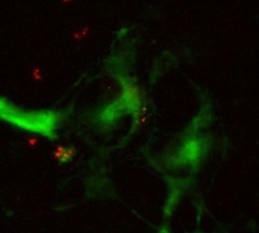

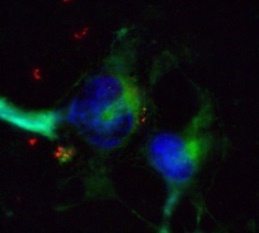


**A ra-DiC16MA**

**N/P=4**


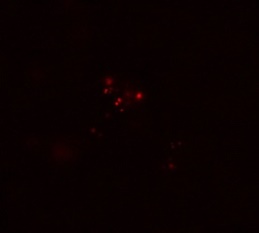

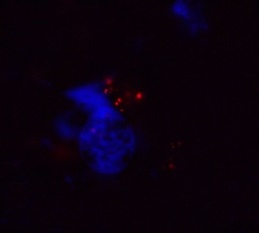

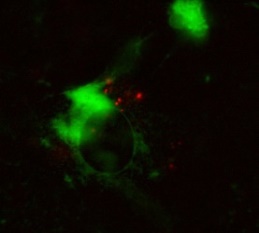

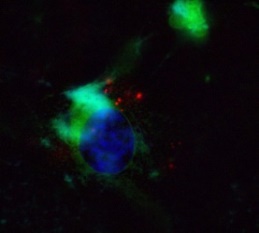


**A ra-DiC16MA**

**N/P=6**


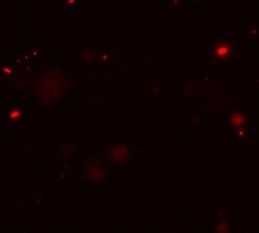

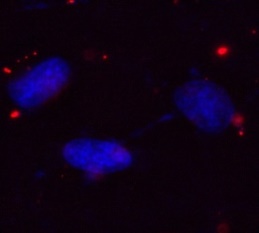

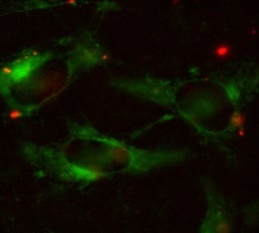

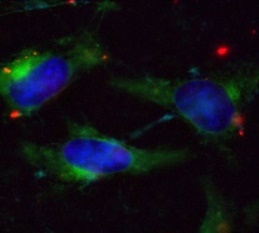


**A ra-DiC16MA**

**N/P=8**


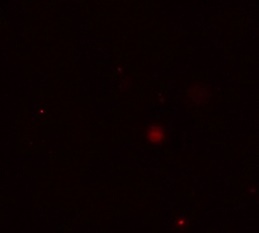

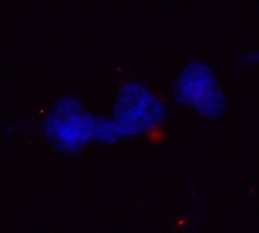

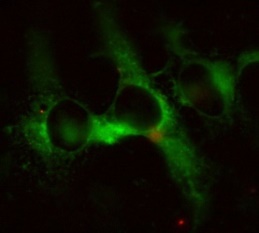

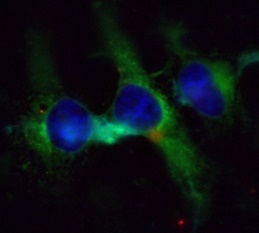


**A ra-DiC16MA**

**N/P=10**


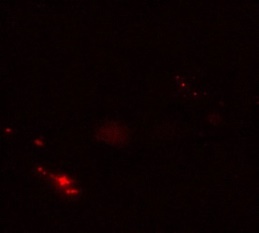

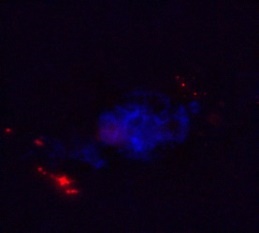

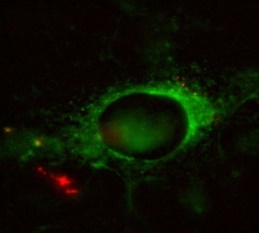

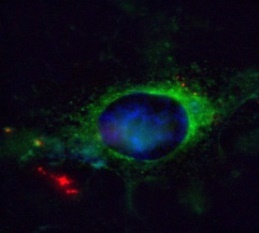


**S5 Fig. Cellular uptake of lipid/pDNA lipoplexes.** Fluorescence microscopic images (100×) of cellular uptake in PC-3 cell (A, Ara-DiC16MA/pDNA complexes), HEK293 cell (B, Ara-DiC14MA/pDNA complexes) and HeLa (C, Ara-DiC16MA/pDNA complexes) at the N/P ratio of 2:1, 4:1, 6:1, 8:1, 10:1 after 4 h of gene transfection (green: Dio used to label cytomembrane, red: Cy3-labeled pDNA, blue: Hochest 33342 stained cell nuclei).
