# Supplementary material for: Synthesis and evaluation of L-arabinose-based cationic glycolipids as effective vectors for pDNA and siRNA in vitro
Source: PLoS One. 2017 Jul 3;12(7):e0180276. doi: 10.1371/journal.pone.0180276 (PMC5495346; doi:10.1371/journal.pone.0180276)
Supplement: S6 Fig — The cell toxicity of HepG2 (A), MCF-7(B) and HeLa(C) treated with cationic lipoplexes (Ara-DiC12MA, Ara-DiC14MA, Ara-DiC16MA, Ara-DiC18MA) at different N/P ratios. The mean cell viability was calculated from three different measurements. Statistical differences from the Lipo2000 are labelled * P < 0.05, ** P < 0.005 and *** P< 0.001. (DOCX) [file pone.0180276.s006.docx]

(B)

(A)

(C)

**S6 Fig. *In vitro* cytotoxicity.** The cell toxicity of HepG2 (A), MCF-7(B) and HeLa(C) treated with cationic lipoplexes (Ara-DiC12MA, Ara-DiC14MA, Ara-DiC16MA, Ara-DiC18MA) at different N/P ratios. The mean cell viability was calculated from three different measurements. Statistical differences from the Lipo2000 are labelled * P < 0.05, ** P < 0.005 and *** P< 0.001.
