# Supplementary material for: Synthesis and evaluation of L-arabinose-based cationic glycolipids as effective vectors for pDNA and siRNA in vitro
Source: PLoS One. 2017 Jul 3;12(7):e0180276. doi: 10.1371/journal.pone.0180276 (PMC5495346; doi:10.1371/journal.pone.0180276)
Supplement: S1 Table — (DOCX) [file pone.0180276.s007.docx]

**S1 Table.** Mean particle size and zeta potential of the lipid/pDNA complexes at different N/P ratios.

| Lipid/pDNA | N/P ratio | Zeta Potential | Average Size | PDI |
| --- | --- | --- | --- | --- |
| Lipid **9a** | 2:1 | -1.7±0.5 | +63.8±4.4 | +0.351±0.010 |
|  | 4:1 | +18.7±1.4 | +87.7±8.0 | +0.220±0.140 |
|  | 6:1 | +24.2±3.6 | +79.6±5.6 | +0.560±0.060 |
|  | 8:1 | +35.0±1.8 | +92.0±7.9 | +0.480±0.040 |
|  | 10:1 | +38.5±3.7 | +84.7±11.5 | +0.410±0.040 |
| Lipid **9b** | 2:1 | +24.2±1.7 | +88.5±5.7 | +0.190±0.055 |
|  | 4:1 | +43.2±2.0 | +98.5±8.0 | +0.330±0.076 |
|  | 6:1 | +39.8±1.8 | +117.0±3.0 | +0.430±0.018 |
|  | 8:1 | +49.1±4.7 | +163.7±1.6 | +0.500±0.067 |
|  | 10:1 | +49.5±0.5 | +176.7±8.8 | +0.526±0.030 |
| Lipid **9c** | 2:1 | -9.4±0.8 | +118.7±1.9 | +0.228±0.050 |
|  | 4:1 | +5.9±0.4 | +182.5±5.5 | +0.080±0.026 |
|  | 6:1 | +32.8±17.4 | +96.5±0.4 | +0.160±0.018 |
|  | 8:1 | +24.1±1.1 | +97.9±1.9 | +0.140±0.007 |
|  | 10:1 | +26.3±1.2 | +84.5±2.4 | +0.521±0.160 |
| Lipid **9d** | 2:1 | -9.2±2.1 | +118.8±8.4 | +0.210±0.045 |
|  | 4:1 | -2.1±0.6 | +133.0±1.1 | +0.130±0.035 |
|  | 6:1 | +22.2±0.5 | +180.2±6.1 | +0.040±0.012 |
|  | 8:1 | +22.3±0.4 | +174.0±2.3 | +0.130±0.037 |
|  | 10:1 | +19.4±1.0 | +127.2±1.7 | +0.090±0.018 |
