# Supplementary material for: Synthesis and evaluation of L-arabinose-based cationic glycolipids as effective vectors for pDNA and siRNA in vitro
Source: PLoS One. 2017 Jul 3;12(7):e0180276. doi: 10.1371/journal.pone.0180276 (PMC5495346; doi:10.1371/journal.pone.0180276)
Supplement: S2 Table — (DOCX) [file pone.0180276.s008.docx]

**S2 Table.** Mean particle size and zeta potential of the lipid/siRNA complexes at different N/P ratios.

| Lipid/siRNA | N/P ratio | Zeta Potential | Average Size | PDI |
| --- | --- | --- | --- | --- |
| Lipid **9a** | 2:1 | +61.8 ± 1.5 | 336.6 ± 6.5 | 0.370 ± 0.037 |
|  | 4:1 | +28.3 ± 3.7 | 334.8 ± 9.1 | 0.472 ± 0.066 |
|  | 6:1 | +52.1 ± 0.3 | 270.8 ± 2.3 | 0.234 ± 0.091 |
|  | 8:1 | +56.2 ± 1.0 | 210.4 ± 25.2 | 0.562 ± 0.042 |
|  | 10:1 | +51.4 ± 0.8 | 318.4 ± 9.3 | 0.305 ± 0.014 |
| Lipid **9b** | 2:1 | +43.6 ± 6.3 | 260.5 ± 13.0 | 0.520 ± 0.047 |
|  | 4:1 | +49.5 ± 2.5 | 266.3 ± 0.5 | 0.270 ± 0.130 |
|  | 6:1 | +50.8 ± 0.6 | 244.5 ±6.3 | 0.366 ± 0.094 |
|  | 8:1 | 68.6 ± 4.3 | 230.4 ± 12.5 | 0.470 ± 0.045 |
|  | 10:1 | +66.2 ± 2.2 | 254.6 ±6.0 | 0.587 ±0.063 |
| Lipid **9c** | 2:1 | +46.2 ± 1.9 | 194.6 ± 6.2 | 0.236 ± 0.034 |
|  | 4:1 | +51.2 ± 0.6 | 191.7 ± 4.3 | 0.201 ± 0.071 |
|  | 6:1 | +54.2 ± 6.0 | 188.7 ± 1.7 | 0.180 ±0.016 |
|  | 8:1 | +46.0 ± 1.9 | 190.1 ± 1.9 | 0.200 ± 0.027 |
|  | 10:1 | +45.5 ± 0.3 | 187.4 ± 1.4 | 0.184 ± 0.025 |
| Lipid **9d** | 2:1 | 39.7 ± 2.3 | 208.6 ± 8.6 | 0.394 ± 0.050 |
|  | 4:1 | +47.7 ± 1.1 | 254.6 ± 5.4 | 0.585 ± 0.083 |
|  | 6:1 | +48.8 ± 2.9 | 309.8 ± 4.5 | 0.540 ± 0.037 |
|  | 8:1 | +44.1 ± 1.4 | 475.3 ± 5.9 | 0.308 ± 0.031 |
|  | 10:1 | +55.7 ± 2.4 | 213.1 ± 6.7 | 0.365 ± 0.011 |
