# Supplementary material for: Synthesis and evaluation of L-arabinose-based cationic glycolipids as effective vectors for pDNA and siRNA in vitro
Source: PLoS One. 2017 Jul 3;12(7):e0180276. doi: 10.1371/journal.pone.0180276 (PMC5495346; doi:10.1371/journal.pone.0180276)
Supplement: S1 File — (DOCX) [file pone.0180276.s009.docx]

**Synthesis of lipid 9a (Ara-DiC12MA)**

**Synthesis of 1,2,3,4-tetra-*O*-acetyl-α,β-L-arabopyranose (1)**

In a 500.0 mL round bottom flask, the acetic anhydride (37.8 mL, 399.7 mmol) was cooled to 0^o^C under stirring. Then perchloric acid (0.2 mL) was added dropwise. The L-arabinose (10.0 g, 66.6 mmol) was added in partition under the temperature was not excess 20^o^C. The reaction mixture was stirring until TLC (petroleum ether : ethyl acetate = 1 : 1) showed the starting material was disappeared, during which time the temperature was gradually raised to ambient temperature. DCM (20.0 mL) was added to it, and then washed with water and CH_2_Cl_2_ for three times. The organic layer was collected, dried over anhydrous sodium sulfate, filtered and the solvent from the filtrate was evaporated on a rotavapor to give crude **1** (20.0 g, 94.3%) as a brown yellow syrup.

**Synthesis of 2,3,4-tri-*O*-acetyl-α,β-L-arabopyranose (2)**

In a 1.0 L round bottom flask, piperazine (5.5 g, 63.8 mmol) was added to the solution of compound **1** **(**18.2 g, 57.2 mmol) in tetrahydrofuran (150.0 mL). The reaction mixture was stirring until TLC (petroleum ether : ethyl acetate = 2 : 1) showed the starting material was almost disappeared, filtered and the solvent from the filtrate was evaporated on a rotavapor. The residue was washed with water and CH_2_Cl_2_. The organic layer was collected, dried over anhydrous sodium sulfate, filtered and concentrated, then purified by silica gel column chromatography with petroleum ether : acetone = 3 : 1 as the eluent to give compound **2** (10.5 g, 66.5%) as a pale yellow syrup.

**Synthesis of 2,3,4-tri-*O*-acetyl-β-L-arabopyranosyl trichloroacetimidate (3)**

In a 500.0 mL round bottom flask, trichloroacetonitrile (35.0 mL, 349.1 mmol) was added to a mixture of the intermediate 2 **(**31.3 g, 113.3 mmol), K_2_CO_3_ (15.7 g, 113.6 mmol) and DBU (1.0 mL, 6.7 mmol) in anhydrous DCM (200.0 mL). The mixture was stirring at room temperature until TLC (petroleum ether : ethyl acetate = 2 : 1) showed the starting material was almost disappeared, filtered and the solvent from the filtrate was evaporated on a rotavapor. The residues was purified by silica gel column chromatography with petroleum ether : ethyl acetate = 4 : 1 as the eluent to give compound **3** (33.6 g, 70.4%) as a white solid.

**Synthesis of 3’-Chloropropyl 2,3,4-tri-*O*-acetyl-α-L-arabopyranoside (4)**

The compound **3** (8.5 g, 20.2 mmol) and 4Ǻ MS (1.5 g) were dried under vacuum for 2h, then 3-chloro-1-propanol (5.5 mL, 65.7 mmol) and dry DCM (150.0 mL) were added. The mixture was stirred and cooled to -20^o^C, and then TMSOTf (75.0 µL, 0.4 mmol) was added under nitrogen atmosphere. The reaction mixture was stirring until TLC (petroleum ether : ethyl acetate = 3 : 1) showed the starting material was disappeared, during which time the temperature was gradually raised to ambient temperature. The mixture was washed with water and CH_2_Cl_2_. The organic layer was collected, dried over anhydrous sodium sulfate, filtered and the solvent from the filtrate was evaporated on a rotavapor. The residues was purified by silica gel column chromatography with petroleum ether : ethyl acetate = 4 : 1 as the eluent to give compound **4** (4.4 g, 62.0%) as a pale yellow syrup. ^1^H NMR (500 MHz, CDCl_3_): δ (ppm): 5.25 (s, 1 H, H-4), 5.17 (t, 1 H, *J*_2,1_ = 7.0 Hz, *J*_2,3_ = 3.0 Hz, H-2), 5.05 (dd, 1 H, *J*_3,2_ = *J*_3,4_ = 3.0 Hz, H-3), 4.42 (d, 1 H, *J*_1,2_ = 7.0 Hz, H-1), 4.03-3.97 (m, 2 H, H-5a, OCH_2_CH_2_CH*H*Cl), 3.66-3.60 (m, 4 H, H-5b, OCH_2_CH_2_CH*H*Cl, OC*H*_2_CH_2_CH_2_Cl), 2.13(s, 3 H, C*H*_3_CO), 2.07 (s, 4 H, C*H*_3_CO, OCH_2_*CH*HCH_2_Cl ), 2.01 (s, 3 H, C*H*_3_CO), 1.99-1.94 (m, 1 H, OCH_2_*CH*HCH_2_Cl), ^13^C NMR (125 MHz, CDCl_3_): δ (ppm): 170.2 (1 C, CH_3_*C*O), 170.0 (1 C, CH_3_*C*O), 169.5 (1 C, CH_3_*C*O), 101.0 (1 C, C-1), 70.0 (1 C, C-3), 69.0 (1 C, C-2), 67.5 (1 C, C-4), 65.7 (1 C, OCH_2_CH_2_*C*H_2_Cl), 63.1 (1 C, C-5), 41.3 (1 C, O*C*H_2_CH_2_CH_2_Cl), 32.0 (1 C, OCH_2_*C*H_2_CH_2_Cl), 20.8 (1 C, *C*H_3_CO), 20.6 (1 C, *C*H_3_CO), 20.5 (1 C, *C*H_3_CO).

**Synthesis of 3’-Azidopropyl 2,3,4-tri-*O*-acetyl-α-L-arabopyranoside (5)**

Sodium azide (7.7 g, 118.4 mmol) was added slowly to a stirred solution of compound 4 (10.4 g, 29.5 mmol) in DMF (150 mL). The reaction mixture was stirring at 75^o^C for 12 h until TLC (petroleum ether : ethyl acetate = 2 : 1) showed the starting material was disappeared, The mixture was washed with water and CH_2_Cl_2_. The organic layer was collected, dried over anhydrous magnesium sulfate, filtered and the solvent from the filtrate was evaporated on a rotavapor. The residues was purified by silica gel column chromatography with petroleum ether : ethyl acetate = 3 : 1 as the eluent to give compound **5** (8.9 g, 84.8% ) as a colourless syrup. ^1^H NMR (500 MHz, CDCl_3_): δ (ppm): 5.23 (dd, 1 H, *J*_4,3_ = *J*_4,5a_ = 3.5 Hz, *J*_4,5b_ = 1.5 Hz, H-4), 5.14 (dd, 1 H, *J*_2,1_ = 7.0 Hz, *J*_2,3_ = 3.5 Hz, H-2), 5.01 (dd, 1 H, *J*_3,2_ = *J*_3,4_ = 3.5 Hz, H-3), 4.38 (d, 1 H, *J*_1,2_ = 7.0 Hz, H-1), 3.99 (dd, 1 H, *J*_5a,4_ = *J*_5a,5b_ = 3.5 Hz, H-5a), 3.92-3.88 (m, 1 H, OCH_2_CH_2_CH*H*N_3_), 3.62 (dd, 1 H, *J*_5b,4_ =1.5 Hz, *J*_5b,5a_ = 3.5 Hz, H-5b), 3.55-3.51 (m, 1H, OCH_2_CH_2_CH*H*N_3_), 3.38-3.31 (m, 2 H, OC*H*_2_CH_2_CH_2_N_3_), 2.09 (s, 3 H, C*H*_3_CO), 2.03 (s, 3 H, C*H*_3_CO), 1.98 (s, 3 H, C*H*_3_CO), 1.88-1.74 (m, 2 H, OCH_2_*CH*_2_CH_2_N_3_), ^13^C NMR (125 MHz, CDCl_3_): δ (ppm): 170.2 (1 C, CH_3_*C*O), 170.0 (1 C, CH_3_*C*O), 169.3 (1 C, CH_3_*C*O), 100.8 (1 C, C-1), 70.0 (1 C, C-3), 69.0 (1 C, C-2), 67.5 (1 C, C-4), 65.8 (1 C,OCH_2_CH_2_*C*H_2_N_3_), 63.1 (1 C, C-5), 47.8 (1 C, O*C*H_2_CH_2_CH_2_N_3_), 28.8 (1 C, OCH_2_*C*H_2_CH_2_N_3_), 20.8 (1 C, *C*H_3_CO), 20.6(2 C, *C*H_3_CO), 20.5 (1 C, *C*H_3_CO).

**Synthesis of 3’-Azidopropyl α-L-arabopyranoside (6)**

Ammonia was bubbled to the solution of compound **5** (8.7 g, 24.2 mmol) in methanol (150.0 mL). The reaction mixture was stirring until TLC (ethyl acetate : methanol = 3 : 1) showed the starting material was disappeared. Then the mixture was concentrated and the residue was purified by silica gel column chromatography with ethyl acetate : methanol = 5 : 1 as the eluent to give compound **6** (4.8 g, 85.7%) as a pale yellow syrup.

**Synthesis of 3’-aminopropyl-α-L-arabopyranoside (7)**

In a 100.0 mL round bottom flask, PPh_3_ (5.7 g, 21.7 mmol) was added to the solution of compound **6** (3.4 g, 14.6 mmol) in THF (40.0 mL) and H_2_O (3.0 mL) under stirring. The reaction mixture was refluxed at 75^o^C, until TLC (ethyl acetate : methanol = 2 : 1) showed the starting material was disappeared. The mixture was concentrated and a white solid was precipitated after water (2.0 mL) was added dropwise to it. Filtered, the mother liquor was evaporated to dryness and dried by vacuum to give yellowish syrup **7** (2.2 g, 72.8%).

**Synthesis of 3’-[(*N*,*N*-di-n-dodecyl)amino]-propyl-α-L-arabopyranoside (8a)**

In a 100.0 mL round bottom flask, anhydrous potassium carbonate (1.8 g, 13.0 mmol) and lauryl bromide (4.2 mL, 17.4 mmol ) was added to the solution of compound **7** (0.9 g, 4.3 mmol) in CH_3_OH (15.0 mL) and CH_3_CH_2_OH (25.0 mL) under stirring. The reaction mixture was refluxed at 70^o^C until TLC (ethyl acetate : methanol = 3 : 1) showed the starting material was disappeared, filtered and the filtrate was evaporated on a rotavapor. The residues was purified by silica gel column chromatography with ethyl acetate : methanol = 5 : 1 as the eluent to give compound **8a** (1.06 g, 44.9%) as a white solid. ^1^H NMR (500 MHz, MeOD):δ (ppm): 4.22 (d, 1 H, *J*_1,2_ = 6.5 Hz, H-1), 3.94-3.88 (m, 1 H, OCH_2_CH_2_CH*H*N(C_12_H_25_)_2_), 3.81-3.76 (m, 2 H, H-3, H-5a), 3.71-3.61 (m, 1 H, OCH_2_CH_2_CH*H*N(C_12_H_25_)_2_), 3.55-3.47 (m, 3 H, H-2, H-4, H-5b), 3.31-3.25 (m, 2 H, OC*H*_2_CH_2_CH_2_N(C_12_H_25_)_2_), 3.12-3.05 (m, 4 H, OCH_2_CH_2_CH_2_N(C*H*_2_CH_2_(CH_2_)_9_CH_3_)_2_ ), 2.03-1.93 (m, 2 H, OCH_2_C*H*_2_CH_2_N(C_12_H_25_)_2_), 1.72-1.1.62 (m, 4 H, OCH_2_CH_2_CH_2_N(CH_2_C*H*_2_ (CH_2_)_9_CH_3_)_2_), 1.38-1.21 (m, 36 H, OCH_2_CH_2_CH_2_N(CH_2_CH_2_(C*H*_2_)_9_CH_3_)_2_), 0.84 (t, 6 H, *J* = 7.0 H_Z_, OCH_2_CH_2_CH_2_N(CH_2_CH_2_(CH_2_)_9_C*H*_3_)_2_); ^13^C NMR (125 MHz, MeOD): δ (ppm): 102.5 (1 C, C-1), 72.0 (1 C, C-4), 70.3 (1 C, C-2), 67.3 (1 C, C-3), 66.3 (1 C, OCH_2_CH_2_*C*H_2_N(C_12_H_25_)_2_), 64.7 (1 C, C-5), 52.3 (2 C, OCH_2_CH_2_CH_2_N(*C*H_2_CH_2_(CH_2_)_9_CH_3_)_2_), 51.0 (1 C, O*C*H_2_CH_2_CH_2_N (C_12_H_25_)_2_), 31.0, 28.7, 28.6, 28.5, 28.4, 28.2, 25.6, 23.2, 22.7, 21.7 (21 C, some signals were overlapped, OCH_2_*C*H_2_CH_2_N(C_12_H_25_)_2_, OCH_2_CH_2_CH_2_N(CH_2_*C*H_2_(CH_2_)_9_CH_3_)_2_, OCH_2_CH_2_CH_2_N (CH_2_CH_2_(*C*H_2_)_9_CH_3_)_2_), 12.4 (2 C, OCH_2_CH_2_CH_2_N(CH_2_CH_2_(CH_2_)_9_*C*H_3_)_2_).

**Synthesis of 3’-[(*N*,*N*-di-n-dodecyl-*N*-methyl) aminonium iodine]-propyl-α-L-arabo pyranoside (9a)**

In a 25.0 mL round bottom flask, the mixture of compound **8a** (300.0 mg, 0.55 mmol) and iodomethane (137.4 μL, 2.20 mmol) in THF (10.0 mL) was stirred at 45 ^o^C until TLC (ethyl acetate : methanol = 3 : 1) showed the starting material was disappeared. The mixture was evaporated to dryness, and then acetone (10.0 mL) was added to the residue. The mixture was cooled with ice bath and a solid was precipitated. The mixture was filtered, and the filter cake was washed with acetone (5.0 mL×3) and dried by vacuum to give white solid **9a** (0.16 g, 42.1%). ^1^H NMR (500 MHz, CDCl_3_):δ (ppm): 4.35 (d, 1 H, *J*_1,2_ = 7.0 Hz, H-1), 4.02-3.90 (m, 3 H, H-3, H-5, OCH_2_CH_2_CH*H*N(CH_3_)(C_12_H_25_)_2_), 3.79-3.59 (m, 6 H, H-2, H-4, H-5a, OCH_2_CH_2_CH*H*N (CH_3_)(C_12_H_25_)_2_, OC*H*_2_CH_2_CH_2_N(CH_3_)(C_12_H_25_)_2_), 3.42-3.29 (m, 4 H, (CH_3_)N(C*H*_2_(CH_2_)_10_CH_3_)_2_), 3.23 (s, 3 H, (C*H*_3_)N(CH_2_(CH_2_)_10_CH_3_)_2_), 2.25-2.10 (m, 2 H, OCH_2_C*H*_2_CH_2_N(CH_3_)(C_12_H_25_)_2_), 1.79-1.62 (m, 4 H, (CH_3_)N(CH_2_C*H*_2_(CH_2_)_9_CH_3_)_2_), 1.38-1.22 (m, 36 H, (CH_3_)N(CH_2_CH_2_ (C*H*_2_)_9_CH_3_)_2_), 0.87 (t, 6 H, *J* = 7.0 Hz, (CH_3_)N(CH_2_CH_2_(CH_2_)_9_C*H*_3_)_2_); ^13^C NMR (125 MHz, CDCl_3_): δ (ppm): 103.3 (1 C, C-1), 72.7, 70.9 (2 C, C-2, C-4), 68.5 (1 C, C-3), 66.6 (1 C, C-5), 65.8 (1 C, OCH_2_CH_2_*C*H_2_N(CH_3_)(C_12_H_25_)_2_), 61.3, 61.1, 60.6 (3 C, O*C*H_2_CH_2_CH_2_N(CH_3_)(C_12_H_25_)_2_, (CH_3_)N(*C*H_2_CH_2_(CH_2_)_9_CH_3_)_2_), 49.5 (1 C, (*C*H_3_)N(C_12_H_25_)_2_), 31.8, 29.6, 29.5, 29.5, 29.4, 29.3, 29.2, 26.3, 23.4, 22.6, 22.5 (21 C, some signals were overlapped, OCH_2_*C*H_2_CH_2_ N(CH_3_)(C_12_H_25_)_2_, (CH_3_)N(CH_2_*C*H_2_(CH_2_)_9_CH_3_)_2_, (CH_3_)N(CH_2_CH_2_(*C*H_2_)_9_CH_3_)_2_), 14.1 (2 C, (CH_3_)N(CH_2_CH_2_(CH_2_)_9_ *C*H_3_)_2_). ESI-MS: m/z =558.5, in agreement with the calculated mass for [M] ^+^ = C_33_H_68_NO_5_^+^.

**Synthesis of lipid 9b (Ara-DiC14MA)**

**Synthesis of 3’-[(*N*,*N*-di-n-myristyl)amino]-propyl-α-L-arabopyranoside (8b)**

In a 100.0 mL round bottom flask, anhydrous potassium carbonate (2.2 g, 15.9 mmol) and myristyl bromide (5.8 mL, 21.2 mmol ) was added to the solution of compound **7** (1.1 g, 5.3 mmol) in CH_3_OH (15.0 mL) and CH_3_CH_2_OH (25.0 mL) under stirring. The reaction mixture was refluxed at 70^o^C until TLC (ethyl acetate : methanol = 3 : 1) showed the starting material was disappeared, filtered and the filtrate was evaporated on a rotavapor. The residues was purified by silica gel column chromatography with ethyl acetate : methanol = 5 : 1 as the eluent to give compound **8b** (1.08 g, 34.0%). ^1^H NMR (500 MHz, MeOD):δ (ppm): 4.28 (d, 1 H, *J*_1,2_ = 6.5 Hz, H-1), 4.01-3.95 (m, 1 H, OCH_2_CH_2_CH*H*N(C_14_H_29_)_2_), 3.87-3.82 (m, 2 H, H-3, H-5a), 3.76-3.71 (m, 1 H, OCH_2_CH_2_CH*H*N(C_14_H_29_)_2_), 3.61-3.55 (m, 3 H, H-2, H-4, H-5b), 3.40-3.34 (m, 2 H, OC*H*_2_CH_2_CH_2_N(C_14_H_29_)_2_), 3.20-3.14 (m, 4 H, OCH_2_CH_2_CH_2_N(C*H*_2_CH_2_(CH_2_)_11_CH_3_)_2_ ), 2.11-2.02 (m, 2 H, OCH_2_C*H*_2_CH_2_N(C_14_H_29_)_2_), 1.81-1.1.71 (m, 4 H, OCH_2_CH_2_CH_2_N(CH_2_C*H*_2_(CH_2_)_11_CH_3_)_2_), 1.42-1.29 (m, 44 H, OCH_2_CH_2_CH_2_N(CH_2_CH_2_(C*H*_2_)_11_CH_3_)_2_), 0.91 (t, 6 H, *J* = 7.0 Hz, OCH_2_CH_2_CH_2_N(CH_2_CH_2_(CH_2_)_11_C*H*_3_)_2_); ^13^C NMR (125 MHz, MeOD): δ (ppm): 102.5 (1 C, C-1), 72.0 (1 C, C-4), 70.3 (1 C, C-2), 67.3 (1 C, C-3), 66.3 (1 C, OCH_2_CH_2_*C*H_2_N(C_14_H_29_)_2_), 64.7 (1 C, C-5), 52.3 (2 C, OCH_2_CH_2_CH_2_N(*C*H_2_CH_2_(CH_2_)_11_CH_3_)_2_), 51.2 (1 C, O*C*H_2_CH_2_CH_2_N(C_14_H_29_)_2_), 31.1, 28.8, 28.7, 28.6, 28.5, 28.4, 28.2, 25.6, 23.1, 22.6, 21.7 (25 C, some signals were overlapped, OCH_2_*C*H_2_CH_2_N(C_14_H_29_)_2_, OCH_2_CH_2_CH_2_N(CH_2_*C*H_2_(CH_2_)_11_CH_3_)_2_, OCH_2_CH_2_CH_2_N(CH_2_CH_2_ (*C*H_2_)_11_CH_3_)_2_), 12.5 (2 C, OCH_2_CH_2_CH_2_N(CH_2_CH_2_(CH_2_)_11_*C*H_3_)_2_).

**Synthesis of 3’-[(*N*,*N*-di-n-myristyl-*N*-methyl)aminonium iodine]-propyl-α-L-arabo pyranoside (9b)**

In a 25.0 mL round bottom flask, the mixture of compound **8b** (280.0 mg, 0.47 mmol) and iodomethane (117.0 μL, 1.88 mmol) in THF (10.0 mL) was stirred at 45 ^o^C until TLC (ethyl acetate : methanol = 3 : 1) showed the starting material was disappeared. The mixture was evaporated to dryness, and then acetone (10.0 mL) was added to the residue. The mixture was cooled with ice bath and a solid was precipitated. The mixture was filtered, and the filter cake was washed with acetone (5.0 mL×3) and dried by vacuum to give white solid **9b** (0.16 g, 45.7%). ^1^H NMR (500 MHz, CDCl_3_):δ (ppm): 4.33 (d, 1 H, *J*_1,2_ = 6.5 Hz, H-1), 4.02-3.90 (m, 3 H, H-3, H-5, OCH_2_CH_2_CH*H*N(CH_3_)(C_14_H_29_)_2_), 3.76-3.58 (m, 6 H, H-2, H-4, H-5a, OCH_2_CH_2_CH*H*N (CH_3_)(C_14_H_29_)_2_, OC*H*_2_CH_2_CH_2_N(CH_3_)(C_14_H_29_)_2_), 3.38-3.29 (m, 4 H, (CH_3_)N(C*H*_2_(CH_2_)_12_CH_3_)_2_), 3.23 (s, 3 H, (C*H*_3_)N(CH_2_(CH_2_)_12_CH_3_)_2_), 2.20-2.10 (m, 2 H, OCH_2_C*H*_2_CH_2_N(CH_3_)(C_14_H_29_)_2_), 1.72-1.64 (m, 4 H, (CH_3_)N(CH_2_C*H*_2_(CH_2_)_11_CH_3_)_2_), 1.37-1.23 (m, 44 H, (CH_3_)N (CH_2_CH_2_ (C*H*_2_)_11_CH_3_)_2_), 0.87 (t, 6 H, *J* = 6.5 Hz, (CH_3_)N(CH_2_CH_2_(CH_2_)_11_C*H*_3_)_2_); ^13^C NMR (125 MHz, CDCl_3_): δ (ppm): 103.3 (1 C, C-1), 72.8, 70.9 (2 C, C-2, C-4), 68.5 (1 C, C-3), 66.5 (1 C, C-5), 65.7 (1 C, OCH_2_CH_2_*C*H_2_N(CH_3_)(C_14_H_29_)_2_), 61.3, 61.2, 60.6 (3 C, O*C*H_2_CH_2_CH_2_N(CH_3_)(C_14_H_29_)_2_, (CH_3_)N(*C*H_2_CH_2_(CH_2_)_11_CH_3_)_2_), 49.5 (1 C, (*C*H_3_)N(C_14_H_29_)_2_), 31.9, 29.7, 29.6, 29.6, 29.5, 29.4, 29.3, 29.2, 26.3, 23.4, 22.6, 22.5 (25 C, some signals were overlapped, OCH_2_*C*H_2_CH_2_ N(CH_3_)(C_14_H_29_)_2_, (CH_3_)N(CH_2_*C*H_2_(CH_2_)_11_CH_3_)_2_, (CH_3_)N(CH_2_CH_2_(*C*H_2_)_11_CH_3_)_2_), 14.1 (2 C, (CH_3_)N(CH_2_CH_2_(CH_2_)_11_*C*H_3_)_2_). ESI-MS: m/z =614.5, in agreement with the calculated mass for [M] ^+^ = C_37_H_76_NO_5_^+^.

**Synthesis of lipid 9c (Ara-DiC16MA)**

**Synthesis of 3’-[(*N*,*N*-di-n-hexadecyl)amino]-propyl-α-L-arabopyranoside (8c)**

In a 100.0 mL round bottom flask, anhydrous potassium carbonate (2.2 g, 15.9 mmol) and hexadecyl bromide (6.5 mL, 21.2 mmol ) was added to the solution of compound **7** (1.1 g, 5.3 mmol) in CH_3_OH (15.0 mL) and CH_3_CH_2_OH (25.0 mL) under stirring. The reaction mixture was refluxed at 70^o^C until TLC (ethyl acetate : methanol = 3 : 1) showed the starting material was disappeared, filtered and the filtrate was evaporated on a rotavapor. The residues was purified by silica gel column chromatography with ethyl acetate : methanol = 5 : 1 as the eluent to give compound **8c** (1.51 g, 43.4%) as a white solid. ^1^H NMR (500 MHz, CDCl_3_): δ (ppm): 4.96, 4.51 (s, 3 H, O*H*), 4.34 (d, 1 H, *J*_1,2_ = 7.0 Hz, H-1), 4.08-3.96 (m, 3 H, H-3, H-5a, OCH_2_CH_2_CH*H*N(C_16_H_33_)_2_), 3.83-3.71 (m, 3 H, H-2, H-4, OCH_2_CH_2_CH*H*N(C_16_H_33_)_2_), 3.62-3.33 (m, 3 H, H-5b, OC*H*_2_CH_2_CH_2_N(C_16_H_33_)_2_), 3.21-3.02 (m, 4 H, OCH_2_CH_2_CH_2_N(C*H*_2_CH_2_ (CH_2_)_13_CH_3_)_2_), 2.21-2.13 (m, 2 H, OCH_2_C*H*_2_CH_2_N(C_16_H_33_)_2_ ), 1.82-1.71 (m, 4 H, OCH_2_CH_2_CH_2_N(CH_2_C*H*_2_(CH_2_)_13_CH_3_)_2_), 1.33-1.21 (m, 52 H, OCH_2_CH_2_CH_2_N(CH_2_CH_2_(C*H*_2_)_13_ CH_3_)_2_), 0.87 (t, 6 H, *J* = 7.0 Hz, OCH_2_CH_2_CH_2_N(CH_2_CH_2_(CH_2_)_13_C*H*_3_)_2_); ^13^C NMR (125 MHz, CDCl_3_): δ (ppm): 103.1 (1 C, C-1), 72.6, 70.7 (2 C, C-2, C-4), 68.5 (1 C, C-3), 66.5, 66.3 (2 C, C-5, OCH_2_CH_2_*C*H_2_N(C_16_H_33_)_2_), 52.2, 51.6 (3 C, O*C*H_2_CH_2_CH_2_N(C_16_H_33_)_2_, OCH_2_CH_2_CH_2_N (*C*H_2_CH_2_(CH_2_)_13_CH_3_)_2_), 31.9, 29.7, 29.6, 29.5, 29.4, 29.3, 29.1, 26.7, 24.4, 23.1, 22.6 (29 C, some signals were overlapped, OCH_2_*C*H_2_CH_2_N(C_16_H_33_)_2_, OCH_2_CH_2_CH_2_N(CH_2_*C*H_2_(CH_2_)_13_CH_3_)_2_, OCH_2_CH_2_CH_2_N(CH_2_CH_2_(*C*H_2_)_13_CH_3_)_2_), 14.1 (2 C, OCH_2_CH_2_CH_2_N(CH_2_CH_2_(CH_2_)_13_*C*H_3_)_2_).

**Synthesis of 3’-[(*N*,*N*-di-n-hexadecyl-*N*-methyl)aminonium iodine]-propyl-α-L-arabo pyranoside (9c)**

In a 25.0 mL round bottom flask, the mixture of compound **8c** (310.0 mg, 0.47 mmol) and iodomethane (117.0 μL, 1.88 mmol) in THF (10.0 mL) was stirred at 45 ^o^C until TLC (ethyl acetate : methanol = 3 : 1) showed the starting material was disappeared. The mixture was evaporated to dryness, and then acetone (10.0 mL) was added to the residue. The mixture was cooled to 0 ^o^C with ice bath and a solid was precipitated. The mixture was filtered, and the filter cake was washed with acetone (5.0 mL×3) and dried by vacuum to give white solid **9c** (0.18 g, 47.4%). ^1^H NMR (500 MHz, CDCl_3_):δ (ppm): 4.32 (d, 1 H, *J*_1,2_ = 4.5 Hz, H-1), 4.05-3.87 (m, 3 H, H-3, H-5a, OCH_2_CH_2_CH*H*N(CH_3_)(C_16_H_33_)_2_), 3.75-3.55 (m, 6 H, H-2, H-4, H-5b, OCH_2_CH_2_CH*H*N (CH_3_)(C_16_H_33_)_2_, OC*H*_2_CH_2_CH_2_N(CH_3_)(C_16_H_33_)_2_), 3.42-3.30 (m, 4 H, (CH_3_)N(C*H*_2_(CH_2_)_14_CH_3_)_2_), 3.25 (s, 3 H, (C*H*_3_)N(CH_2_(CH_2_)_14_CH_3_)_2_), 2.27-2.11 (m, 2 H, OCH_2_C*H*_2_CH_2_N(CH_3_)(C_16_H_33_)_2_), 1.79-1.65 (m, 4 H, (CH_3_)N(CH_2_C*H*_2_(CH_2_)_13_CH_3_)_2_), 1.38-1.23 (m, 52 H, (CH_3_)N(CH_2_CH_2_ (C*H*_2_)_13_CH_3_)_2_), 0.87 (t, 6 H, *J* = 7.0 Hz, (CH_3_)N(CH_2_CH_2_ (CH_2_)_13_C*H*_3_)_2_); ^13^C NMR (125 MHz, CDCl_3_): δ (ppm): 103.6 (1 C, C-1), 72.7, 70.7 (2 C, C-2, C-4), 68.6 (1 C, C-3), 66.9 (1 C, C-5), 65.8 (1 C, OCH_2_CH_2_*C*H_2_N(CH_3_)(C_16_H_33_)_2_), 61.2 (2 C, (CH_3_)N(*C*H_2_ (CH_2_)_14_CH_3_)_2_,), 60.5 (1 C, O*C*H_2_CH_2_CH_2_N(CH_3_)(C_16_H_33_)_2_), 49.4 (1 C, (*C*H_3_)N(C_16_H_33_)_2_), 31.9, 29.7, 29.6, 29.6, 29.5, 29.3, 29.2, 26.3, 23.5, 22.6, 22.5 (29 C, some signals were overlapped, OCH_2_*C*H_2_CH_2_N(CH_3_)(C_16_H_33_)_2_, (CH_3_)N(CH_2_*C*H_2_(CH_2_)_13_CH_3_)_2_, (CH_3_)N (CH_2_CH_2_(*C*H_2_)_13_CH_3_)_2_), 14.1 (2 C, (CH_3_)N(CH_2_CH_2_ (CH_2_)_13_*C*H_3_)_2_). ESI-MS: m/z =699.18, in agreement with the calculated mass for [M] ^+^ = C_43_H_88_NO_5_^+^.

**Synthesis of lipid 9d (Ara-DiC18MA)**

**Synthesis of 3’-[(*N*,*N*-di-n-octadecyl)amino]-propyl-α-L-arabopyranoside (8d)**

In a 100.0 mL round bottom flask, anhydrous potassium carbonate (3.4 g, 24.6 mmol) and octadecy bromide (7.1 mL, 32.8 mmol ) was added to the solution of compound **7** (1.7 g, 8.2 mmol) in CH_3_OH (15.0 mL) and CH_3_CH_2_OH (25.0 mL) under stirring. The reaction mixture was refluxed at 70^o^C until TLC (ethyl acetate : methanol = 3 : 1) showed the starting material was disappeared, filtered and the filtrate was evaporated on a rotavapor. The residues was purified by silica gel column chromatography with ethyl acetate : methanol = 5 : 1 as the eluent to give compound **8d** (2.1 g, 36.1%) as a white solid. ^1^H NMR (500 MHz, MeOD):δ (ppm): 4.27 (d, 1 H, *J*_1,2_ = 6.5 Hz, H-1), 3.99-3.94 (m, 1 H, OCH_2_CH_2_CH*H*N(C_18_H_37_)_2_), 3.86-3.83 (m, 2 H, H-3, H-5a), 3.74-3.70 (m, 1 H, OCH_2_CH_2_CH*H*N(C_18_H_37_)_2_), 3.59-3.52 (m, 3 H, H-2, H-4, H-5b), 3.35-3.33 (m, 2 H, OC*H*_2_CH_2_CH_2_N(C_18_H_37_)_2_), 3.17-3.13 (m, 4 H, OCH_2_CH_2_CH_2_N(C*H*_2_CH_2_(CH_2_)_15_CH_3_)_2_), 2.06-2.03 (m, 2 H, OCH_2_C*H*_2_CH_2_N(C_18_H_37_)_2_), 1.73-1.71 (m, 4 H, OCH_2_CH_2_CH_2_N(CH_2_C*H*_2_ (CH_2_)_15_CH_3_)_2_), 1.38-1.28 (m, 60 H, OCH_2_CH_2_CH_2_N(CH_2_CH_2_(C*H*_2_)_15_CH_3_)_2_), 0.91 (t, 6 H, *J* = 7.0 Hz, OCH_2_CH_2_CH_2_N(CH_2_CH_2_(CH_2_)_15_C*H*_3_)_2_); ^13^C NMR (125 MHz, MeOD): δ (ppm): 104.5 (1 C, C-1), 74.0 (1 C, C-4), 72.3 (1 C, C-2), 69.3 (1 C, C-3), 68.3 (1 C, OCH_2_CH_2_*C*H_2_N(C_18_H_37_)_2_), 66.7 (1 C, C-5), 54.3 (2 C, OCH_2_CH_2_CH_2_N(*C*H_2_CH_2_(CH_2_)_15_CH_3_)_2_), 53.2 (1 C, O*C*H_2_CH_2_CH_2_N (C_18_H_37_)_2_), 33.1, 30.8, 30.7, 30.6, 30.5, 30.4, 30.2, 27.6, 25.2, 24.6, 23.7 (33 C, some signals were overlapped, OCH_2_*C*H_2_CH_2_N(C_18_H_37_)_2_, OCH_2_CH_2_CH_2_N(CH_2_*C*H_2_(CH_2_)_15_CH_3_)_2_, OCH_2_CH_2_CH_2_N (CH_2_CH_2_(*C*H_2_)_15_CH_3_)_2_), 14.5 (2 C, OCH_2_CH_2_CH_2_N(CH_2_CH_2_(CH_2_)_15_*C*H_3_)_2_).

**Synthesis of 3’-[(*N*,*N*-di-n-octadecyl-*N*-methyl)aminonium iodine]-propyl-α-L-arabo pyranoside (9d)**

In a 25.0 mL round bottom flask, the mixture of compound **8d** (300.0 mg, 0.42 mmol) and iodomethane (110.0 μL, 1.68 mmol) in THF (10.0 mL) was stirred at 45 ^o^C until TLC (ethyl acetate : methanol = 3 : 1) showed the starting material was disappeared. The mixture was evaporated to dryness, and then acetone (10.0 mL) was added to the residue. The mixture was cooled to 0 ^o^C with ice bath and a white solid was precipitated. The mixture was filtered, and the filter cake was washed with cooled acetone (5.0 mL×3) and dried by vacuum to give **9d** (0.2 g, 52.8%). ^1^H NMR (500 MHz, CDCl_3_):δ (ppm): 4.31 (d, 1 H, *J*_1,2_ = 6.0 Hz, H-1), 4.05-3.95 (m, 3 H, H-3, H-5, OCH_2_CH_2_CH*H*N(CH_3_)(C_18_H_37_)_2_), 3.79-3.59 (m, 7.0 H, H-2, H-4, H-5a, OCH_2_CH_2_CH*H*N (CH_3_)(C_18_H_37_)_2_, OC*H*_2_CH_2_CH_2_N(CH_3_)(C_18_H_37_)_2_), 3.42-3.29 (m, 4 H, (CH_3_)N(C*H*_2_(CH_2_)_16_CH_3_)_2_), 3.24 (s, 3 H, (C*H*_3_)N(CH_2_(CH_2_)_16_CH_3_)_2_), 2.22-2.09 (m, 2 H, OCH_2_C*H*_2_CH_2_N(CH_3_)(C_18_H_37_)_2_), 1.75-1.64 (m, 4 H, (CH_3_)N(CH_2_C*H*_2_(CH_2_)_15_CH_3_)_2_), 1.39-1.22 (m, 60 H, (CH_3_)N (CH_2_CH_2_ (C*H*_2_)_15_CH_3_)_2_), 0.87 (t, 6 H, *J* = 7.0 Hz, (CH_3_)N(CH_2_CH_2_(CH_2_)_15_C*H*_3_)_2_); ^13^C NMR (125 MHz, CDCl_3_): δ (ppm): 103.4 (1 C, C-1), 72.9, 70.9 (2 C, C-2, C-4), 68.5 (1 C, C-3), 66.5 (1 C, C-5), 65.7 (1 C, OCH_2_CH_2_*C*H_2_N(CH_3_)(C_18_H_37_)_2_), 61.3, 60.6 (3 C, O*C*H_2_CH_2_CH_2_N(CH_3_)(C_18_H_37_)_2_, (CH_3_)N(*C*H_2_CH_2_(CH_2_)_15_CH_3_)_2_), 49.4 (1 C, (*C*H_3_)N(C_18_H_37_)_2_), 31.9, 29.7, 29.6, 29.5, 29.4, 29.3, 29.2, 26.3, 23.4, 22.6, 22.5 (33 C, some signals were overlapped, OCH_2_*C*H_2_CH_2_ N(CH_3_)(C_18_H_37_)_2_, (CH_3_)N(CH_2_*C*H_2_(CH_2_)_15_CH_3_)_2_, (CH_3_)N(CH_2_CH_2_(*C*H_2_)_15_CH_3_)_2_), 14.1 (2 C, (CH_3_)N(CH_2_CH_2_ (CH_2_)_11_*C*H_3_)_2_). ESI-MS: m/z =726.7, in agreement with the calculated mass for [M] ^+^ = C_45_H_92_NO_5_^+^.
