# Supplementary figures and images for: Synthesis and evaluation of L-arabinose-based cationic glycolipids as effective vectors for pDNA and siRNA in vitro
Source: PLoS One. 2017 Jul 3;12(7):e0180276. doi: 10.1371/journal.pone.0180276 (PMC5495346; doi:10.1371/journal.pone.0180276)

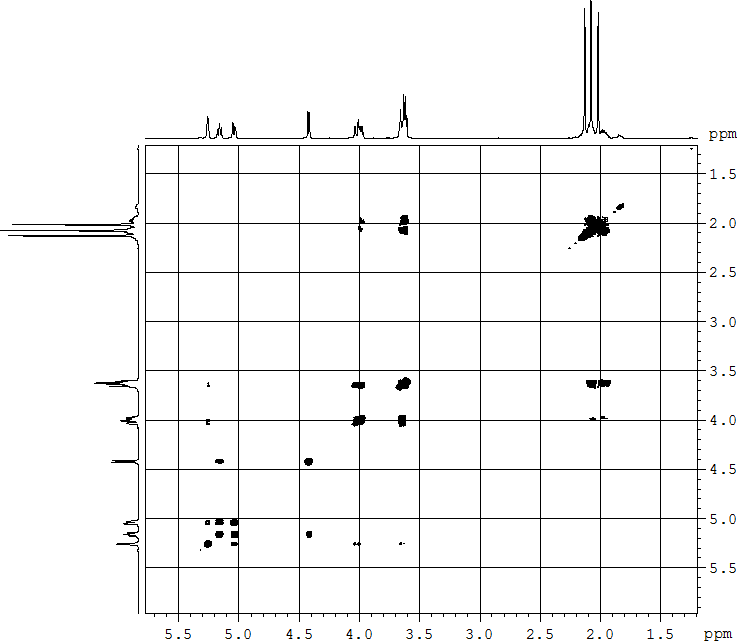


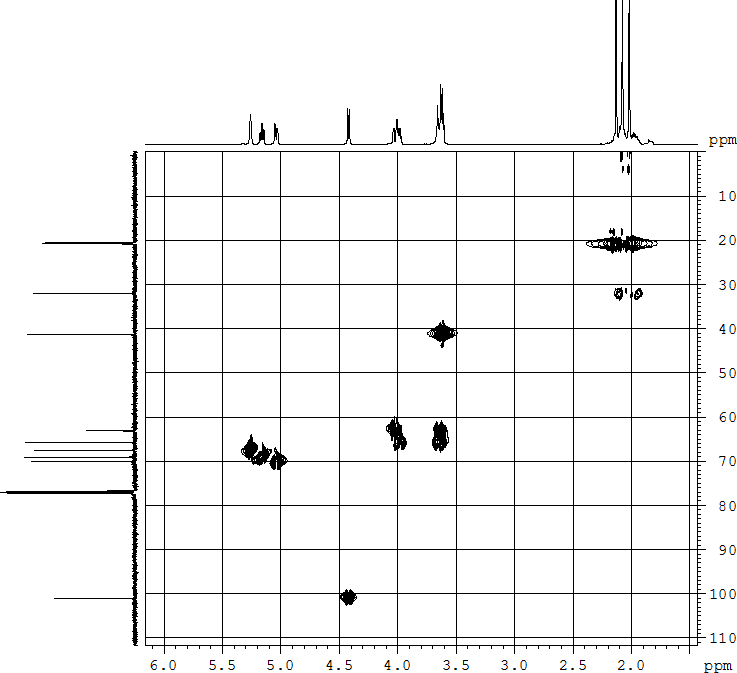


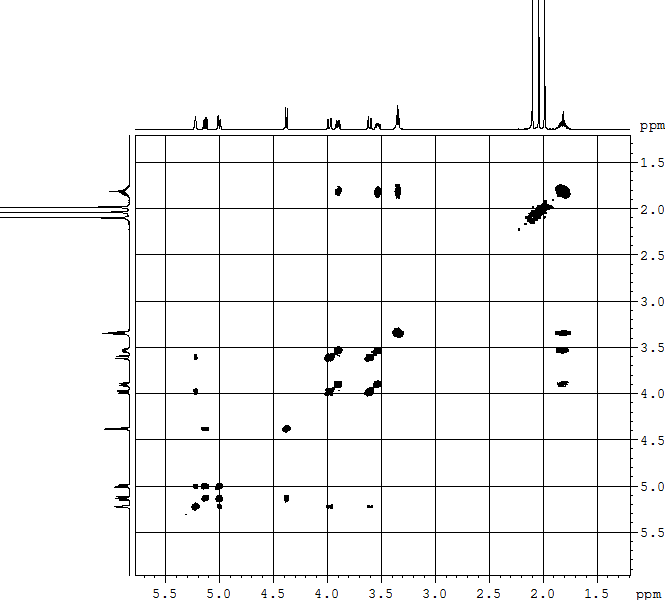


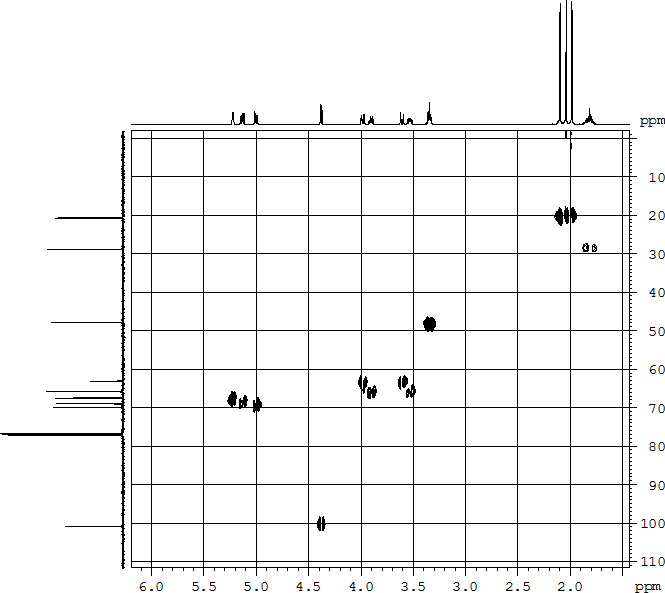


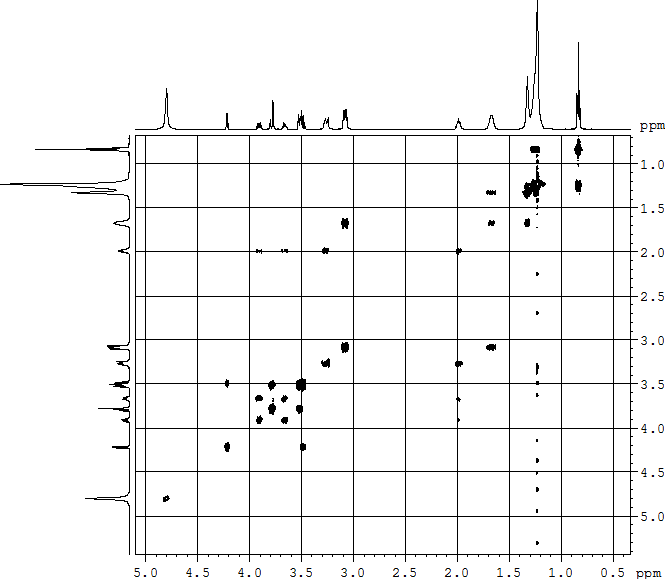


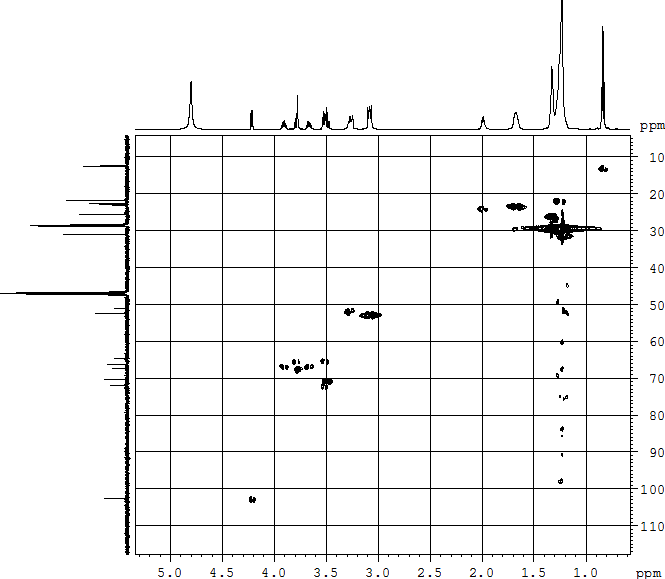


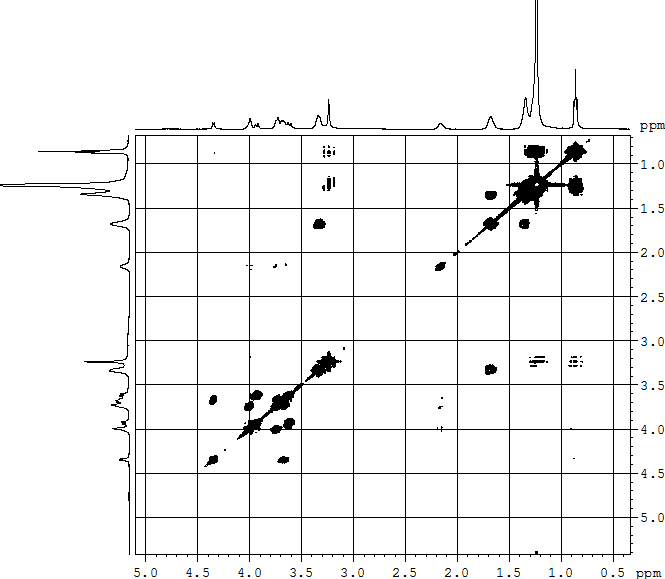


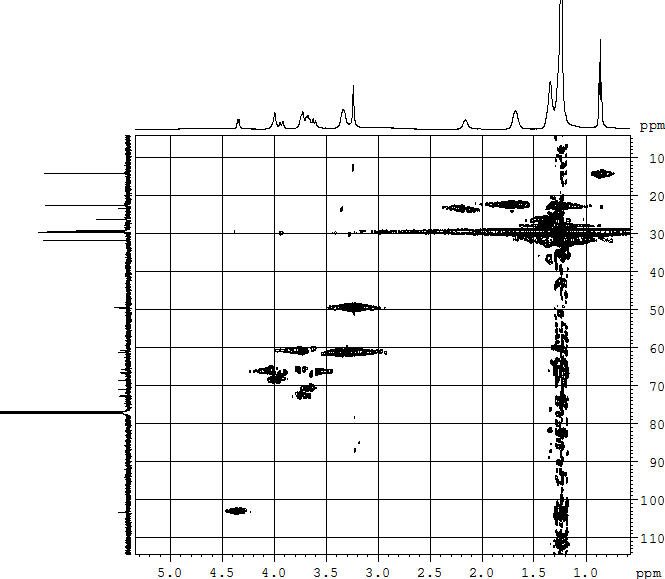


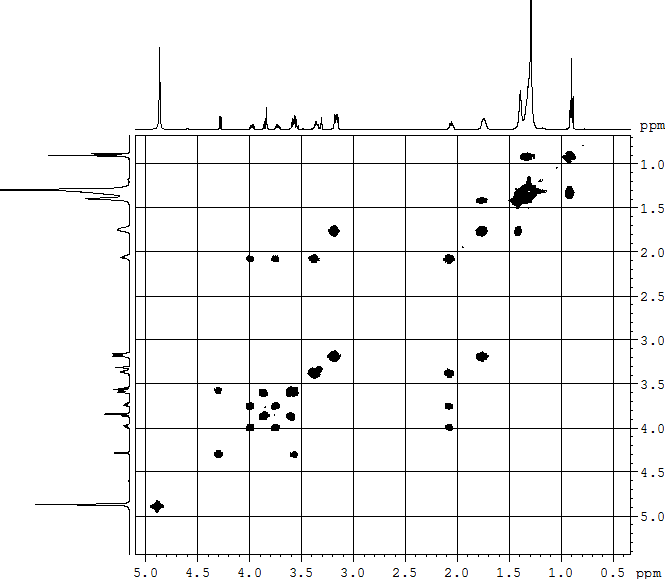


**
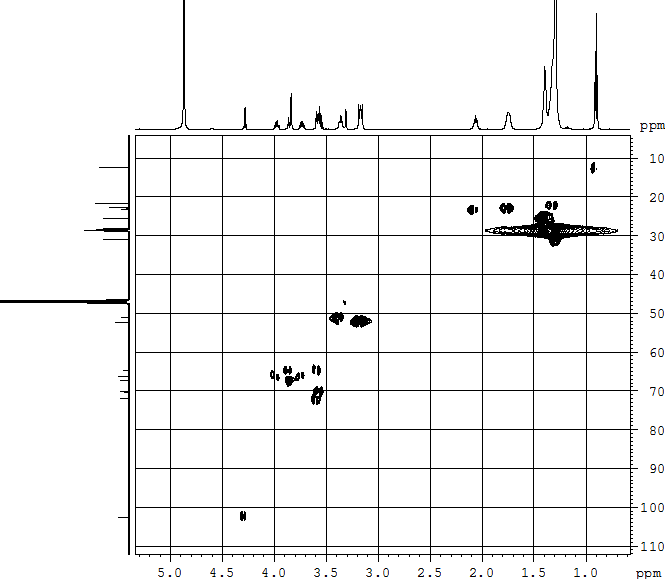
**


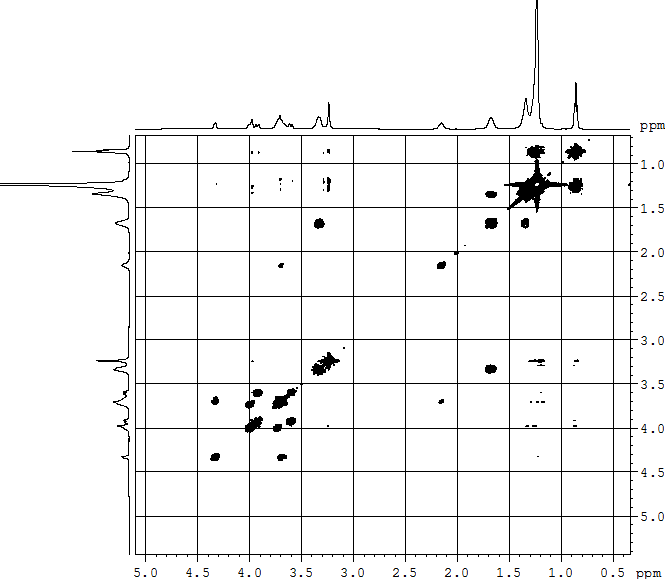


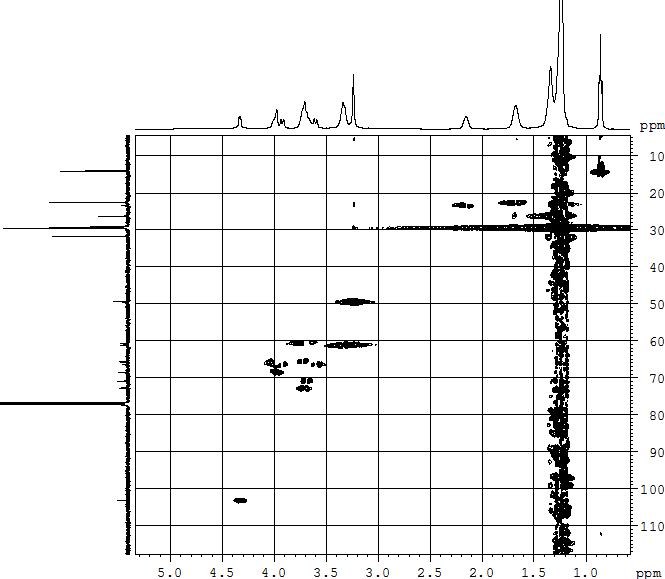


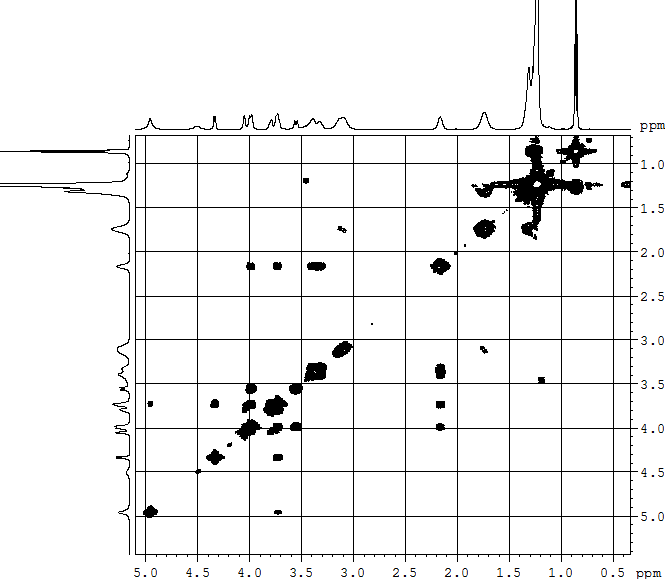


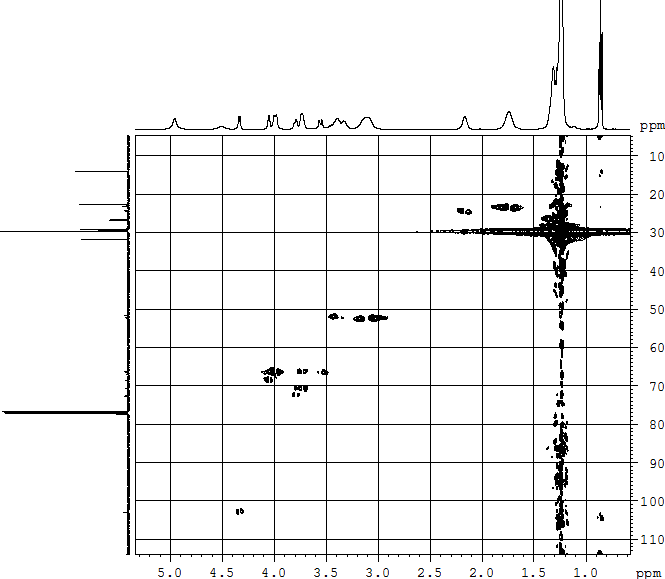


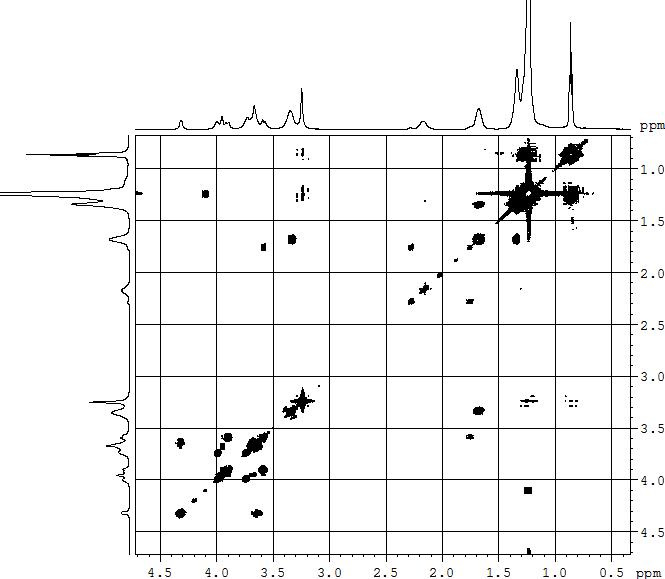


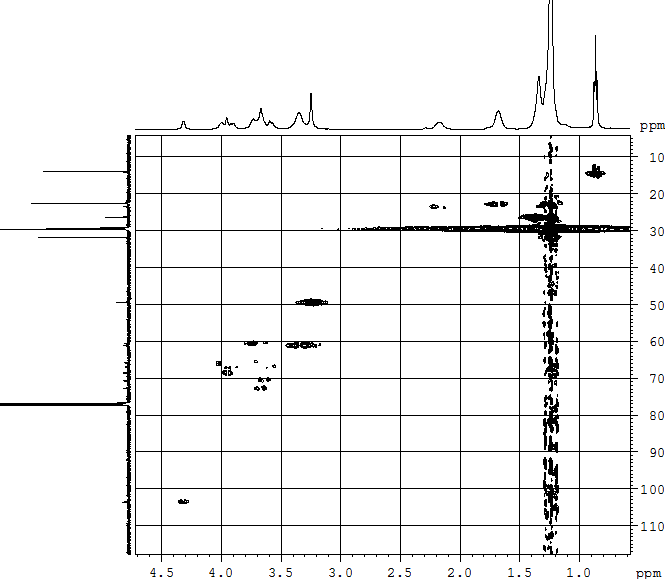


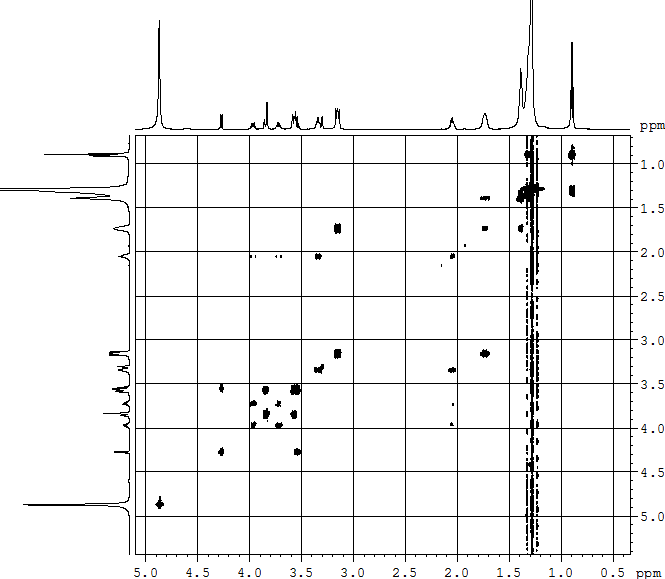


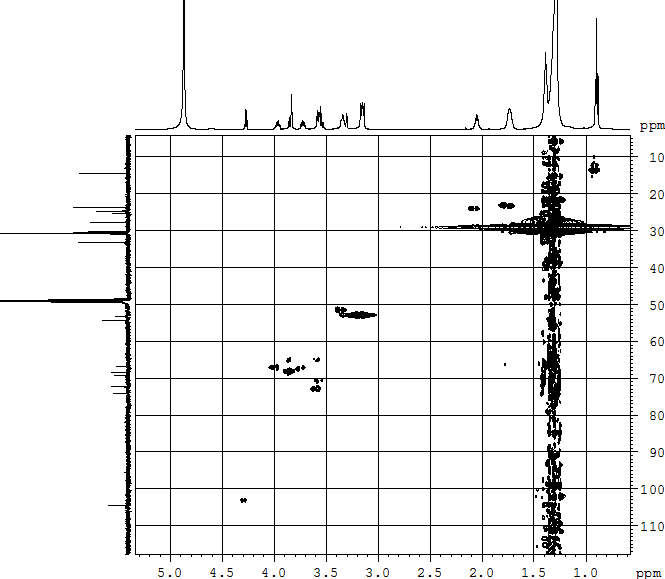


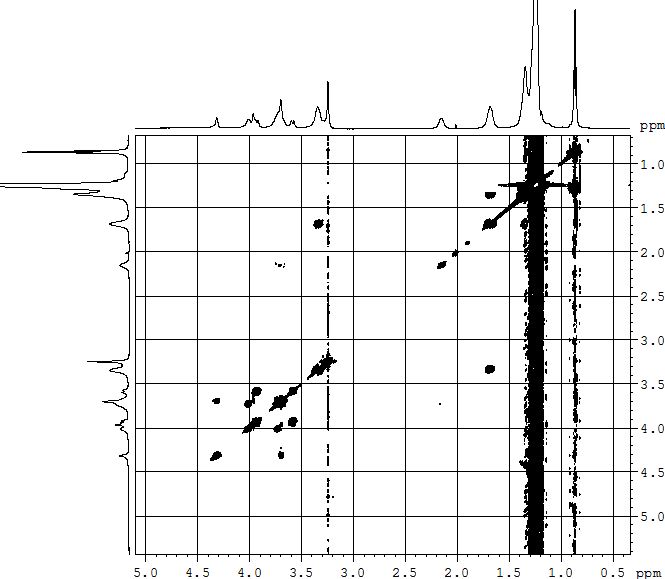


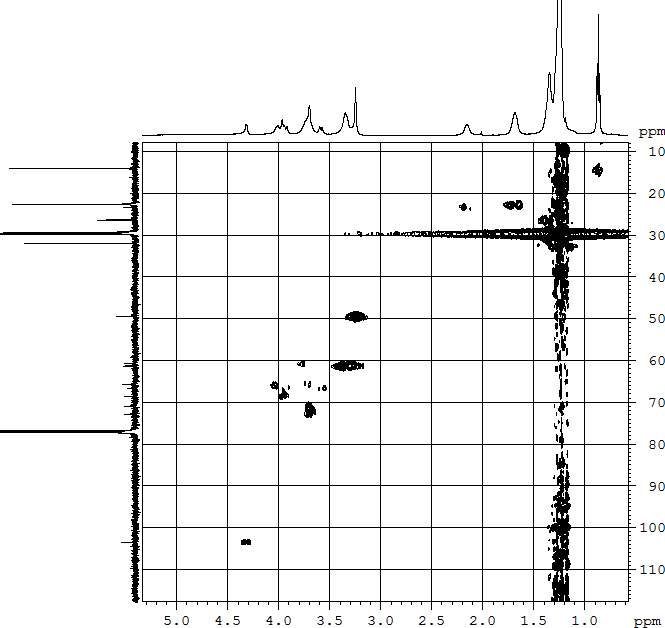

Supplement: S2 File — (DOCX) [file pone.0180276.s010.docx]
